# Trophic niche shifts and phenotypic trait evolution are largely decoupled in Australasian parrots

**DOI:** 10.1186/s12862-021-01940-4

**Published:** 2021-11-27

**Authors:** Vicente García-Navas, Joseph A. Tobias, Manuel Schweizer, Daniel Wegmann, Richard Schodde, Janette A. Norman, Les Christidis

**Affiliations:** 1 Department of Integrative Ecology, Doñana Biological Station EBD (CSIC), Seville, Spain; 2 Department of Evolutionary Biology and Environmental Studies, University of Zurich, Zurich, Switzerland; 3 Centre for Ecology, Evolution and Environmental Changes (cE3c), University of Lisbon, Lisbon, Portugal; 4 Department of Life Sciences (Silwood Park), Faculty of Natural Sciences, Imperial College London, London, UK; 5 Natural History Museum of Bern, Bern, Switzerland; 6 Department of Biology, University of Fribourg, Fribourg, Switzerland; 7 Swiss Institute of Bioinformatics, Fribourg, Switzerland; 8 Australian National Wildlife Collection, CSIRO Sustainable Ecosystems, Canberra, Australia; 9 Southern Cross University, Coffs Harbour, NSW Australia

**Keywords:** Adaptive landscape, Beak morphology, Diet, Ecomorphology, Evolutionary jump, Feeding ecology, Nectarivory, Psittaculidae

## Abstract

**Background:**

Trophic shifts from one dietary niche to another have played major roles in reshaping the evolutionary trajectories of a wide range of vertebrate groups, yet their consequences for morphological disparity and species diversity differ among groups.

**Methods:**

Here, we use phylogenetic comparative methods to examine whether the evolution of nectarivory and other trophic shifts have driven predictable evolutionary pathways in Australasian psittaculid parrots in terms of ecological traits such as body size, beak shape, and dispersal capacity.

**Results:**

We found no evidence for an ‘early-burst’ scenario of lineage or morphological diversification. The best-fitting models indicate that trait evolution in this group is characterized by abrupt phenotypic shifts (evolutionary jumps), with no sign of multiple phenotypic optima correlating with different trophic strategies. Thus, our results point to the existence of weak directional selection and suggest that lineages may be evolving randomly or slowly toward adaptive peaks they have not yet reached.

**Conclusions:**

This study adds to a growing body of evidence indicating that the relationship between avian morphology and feeding ecology may be more complex than usually assumed and highlights the importance of adding more flexible models to the macroevolutionary toolbox.

**Supplementary Information:**

The online version contains supplementary material available at 10.1186/s12862-021-01940-4.

## Background

Ecological opportunity—defined as newly available resources associated with the colonization of unoccupied areas (e.g., new islands; [1]) or the invasion of previously inaccessible niches [2]—can act as a catalyst boosting lineage and phenotypic diversification. Rapid lineage accumulation and ecological diversification can also arise through the extinction of a previously dominant group (i.e., release from competition) and/or the appearance of a key innovation that allows a lineage to interact with the environment in a novel way [3–5]. For instance, the emergence of the New Guinean cordillera, the spread of arid habitats in the Australian continent, and the extinction of thylacinids may have spurred the diversification of dasyurid marsupials [6], whereas the evolution of physiological traits associated with tolerance to arid conditions may be responsible for the exceptionally high diversification rates observed in some lineages of Australian squamate reptiles like scincid lizards or pygopodoid geckos (e.g., [7]). A hallmark of this process—related to the concept of adaptive radiation—is the existence of a signature consisting of an ‘early-burst’ of speciation and morphological divergence via niche filling, followed by a subsequent slowdown in the rate of diversification [8]. Classic adaptive radiations seem to be uncommon in birds, especially on continents, where allopatric diversification occurs more frequently than on island systems [9]. Most continental clades that represent cases of rapid speciation show relatively subtle ecomorphological differentiation among all species, and are thus often regarded as “non-adaptive radiations” (e.g. [10, 11]).

Non-adaptive radiations arise through processes that are unrelated to divergent niche exploitation, whereas adaptive radiations are driven by ecological diversification that confers individuals an advantage in terms of resource use [12]. There are two main pathways by which changes in ecology or behaviour can be successful and promote adaptive radiation. One involves the removal of previous constraints, creating the opportunity for further diversification that enables new adaptations; the other pulls characters towards a new optimal phenotype at the population level [13, 14]. Both possibilities are connected with the concept of the Simpsonian Adaptive Landscape ([15]; see also [16]), a theoretical framework revitalized in recent years with the advent of numerous methodological tools specifically aimed at inferring ‘multi-peak’ landscapes [17–19]. The so-called macroevolutionary landscapes depict the movements of adaptive peaks over million-year time scales [20]. Consequently, it is now feasible to ascertain the influence of ecological shifts in generating morphological diversity across radiations over a multivariate phenotype space where new species aggregate into ever more densely occupied regions of ecological niche space (e.g., [21]). These transitions between states are often thought to occur in a non-gradualist manner, in a regime that Simpson termed “quantum evolution”, whereby extended periods of evolutionary stasis are punctuated by short pulses of rapid change [15].

Among the plethora of ecological shifts, changes in diet have been suggested to play a key role in triggering phenotypic divergence across a range of bird clades (e.g., [22, 23]). Most adaptive radiations including textbook examples like Madagascan vangas [24], are associated with the expansion of total niche breadth beyond that of the ancestral range. That is, a shift from generalist habits to more specialized dietary modes and vice versa (see e.g., [25]). Hence, adaptation to nectarivory by some clades of birds (and bats) may have constituted a stimulus for diversification [26]. Nectarivorous birds have developed a variety of adaptations in order to exploit this sugar-rich, liquid food source produced by plants and which supplies abundant energy to those species that are able to harvest it (see [26] and references therein). Morphological adaptations include changes in body size, beak, and tongue structure, as well as modifications in the digestive tract [27]. Accordingly, New World nectarivorous hummingbirds (family Trochilidae) and some Old World passerine nectarivores including the sunbirds (Nectariniidae), the Hawaiian honeycreepers (Fringillidae) and the Australian honeyeaters (Meliphagidae) are frequently evoked as paradigmatic examples of convergent evolution to a common evolutionary niche [28]. Members of these families share some key traits viewed as adaptations to nectarivory, including elongated bills and extensile, brush-tipped tongues. In a recent study, [29] concluded that the evolution of nectarivory among honeyeaters has had important consequences for both rates of body size evolution and morphological adaptations (bill size and shape) in this family. The diversification of honeyeaters may have constrained the subsequent radiation of Australasian psittaculid parrots, many of whose members also exhibit a diet rich in nectar [29]. The influence of a more recent shift to a nectarivorus diet in psittaculid parrots appears to have had a more limited impact in terms of phenotypic disparity [30, 31], yet this has not been formally tested so far.

In the present study, we examined the evolutionary impact of dietary specialization on the level of morphological and lineage diversification observed among 117 species of Old-World parrots (family Psittaculidae) inhabiting the Australasian region. This family includes lories and lorikeets (Loriini), the most speciose tribe of Old-World parrots and whose rapid diversification may be linked to the predominance of nectarivory in this clade [27, 31, 32]. Nectarivory, however, is not exclusive to this tribe, and species belonging to other tribes of Australasian parrots also exhibit this specialized diet. Nectarivorous parrots feed at open, cup-like, or shaving-brush flowers produced by trees and shrubs, with an overwhelming preference for eucalypts [33]. Here, we first explored the relationship between morphology and dietary strategy (five levels comprising nectarivory and four other categories: frugivory, herbivory, granivory, and omnivory). We also addressed whether the adaptation to nectarivory has led to changes in phenotype and beak morphology in this family and whether the macroevolutionary dynamics of trait evolution in nectarivorous lineages are decoupled from those of non-nectarivorous lineages. We would expect the existence of segregation in morphological space between nectarivorous species and those with less specialized or ancestral feeding habits such as omnivory and granivory, which should exhibit a generalist morphology that allows them to exploit different feeding resources. Specifically, we would expect that nectar-dependent parrots exhibit slender (and structurally weaker) beaks than those of similar-sized granivorous parrots, reflecting their reduced dependence on hard foodstuffs. Since nectar constitutes a scarcer and more seasonal resource than seeds or insects, and nectarivorous species have to travel large distances searching for flushes of flowering, we also predict that parrots relying heavily on nectar should exhibit a higher dispersal capacity than other more generalist species. In turn, a diet shift to nectarivory may have enabled lories to colonize many islands in the Australasian region whether this change is associated with an improvement of the species’ ability to disperse. Nevertheless, a high dispersal ability could also be highly beneficial for species of arid habitats which, in some cases, have a rather nomadic lifestyle and have to follow areas with recent rainfall. Since the radiation of psittaculid parrots span a huge geographic area (mainland Australia, Papua New Guinea, and surrounding islands) and a time period including major climatic shifts and geological events (aridification of the Australian continent, uplift of the Huon Peninsula), we would expect that this radiation has been shaped by recurrent events of ecological opportunity over time due to dynamic habitat and range changes rather than by ecological opportunity at the beginning of its evolutionary history. Thus, we do not expect a diversity-dependent signature of species accumulation over time as predicted by the niche-filling nature of adaptive radiations in this family [4, 5, 8].

## Results

**Fig. 1:**
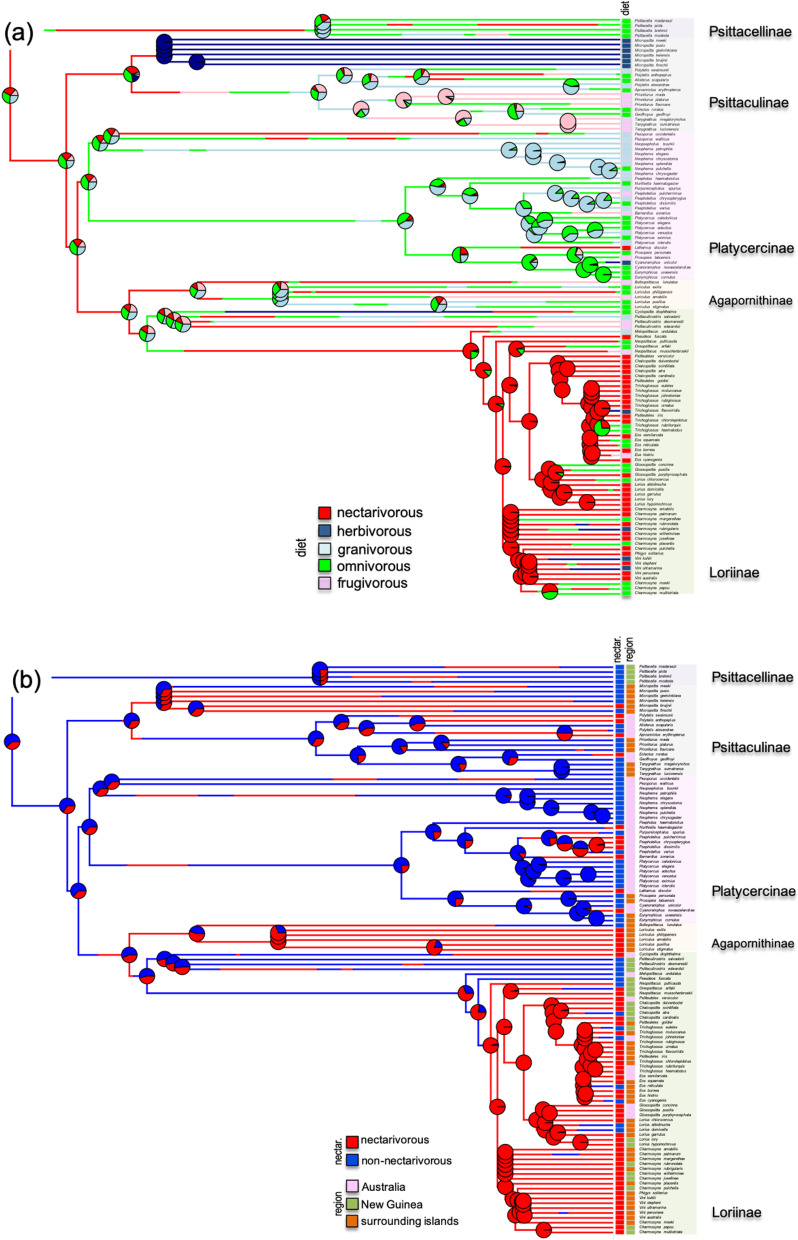
Stochastic character-mapped SIMMAP reconstructions of (**a**) diet (five dietary regimes) and (**b**) nectarivory (yes/no) in Australasian parrots. Species are coded by discrete trophic designation (diet: green = omnivore; red = nectarivore; light blue = granivore; dark blue = herbivore; pink = frugivore; nectarivory: blue = no; red = yes) and region to which they belong to (Australia = pink; New Guinea = green; surrounding islands = orange). One of the SIMMAP reconstructions picked at random is shown. Pie charts at nodes indicate proportion of discrete dietary states reconstructed at the node from 500 simulations using the symmetric-rates (SYM) and equal-rates (ER) model, respectively

Members of the two most species-rich families, Loriinae and Platycercinae, are distributed among all diet categories. Our SIMMAP analysis indicated that there is no unambiguous support for one dietary niche or another for the most recent common ancestor (MRCA) of psittaculid parrots from the Australasian region (Fig. 1). See also Additional file 1: Figs. S1, S2.

### Lineage diversification

**Fig. 2:**
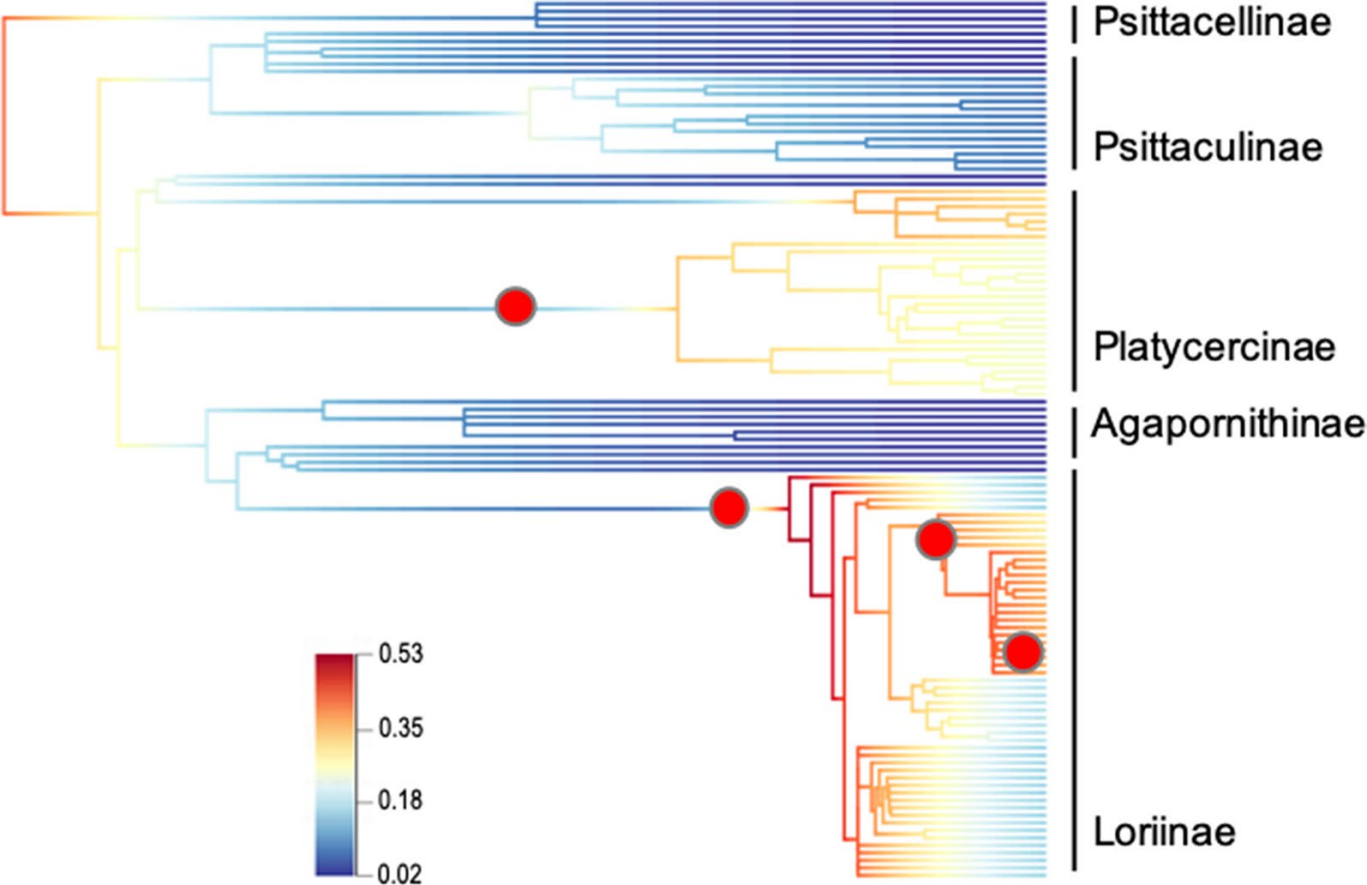
Rate-shifts in diversification of psittaculid parrots estimated from BAMM analysis. Red circles indicate locations of shift configurations in net diversification rate. The coloured section of each branch represents the mean of the posterior density of diversification rate with cold and hot areas indicating low and high diversification rates, respectively

Our BAMM analysis yielded a general pattern of decreasing rates over time in Psitacellinae, Psittaculinae, and Agapornithinae and higher diversification rates in Platycercinae (one shift) and particularly in Loriinae (three shifts) (Fig. 2). We found no support for an early-burst of diversification in Australasian parrots.

### Disparity-through-time plots

**Fig. 3:**
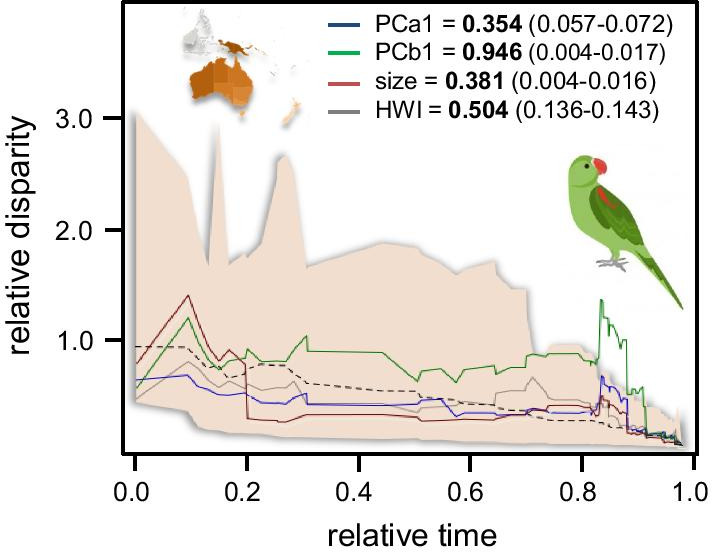
Disparity-through-time (DTT) plots for PCa1 (blue line), PCb1(green line), body size (red line) and HWI (grey line). Disparity along the *y*-axis is the average subclade disparity divided by total clade disparity and is calculated at each internal node of the tree. The dotted lines represent values of trait disparity expected under BM by simulating trait evolution 2500 times each across the tree. The shaded area denotes the 95% range of the simulated data. For relative time values 0 represents the root and 1 the tip of the phylogeny. MDI values along the range of *p*-values computed by means of the rank envelope test [73] are given

The output of DTT analyses showed a pattern consistent with a non-adaptive diversification during the clades’ history (Fig. 2c). Within-clade disparity was higher than between clades, mostly in the recent history (~ 6 Mya) of this group (Fig. 3). All traits showed MDI values > 0.350 and deviation from the null (greater within-clade disparity) was significant for PCb1 and size (Fig. 3). High values of MDI reflect that subclades occupied a greater than expected proportion of morphospace suggesting the existence of clustering (i.e., overlapping morphotypes). Interestingly, we found no support for convergent evolution driving this pattern using SURFACE (unpubl. material).

### Trait model fitting

Results from model fitting are summarized in Table 1, which provides information regarding fit (log-likelihoods and AIC_c_ values) and parameter estimates for each of the evolutionary models (averaged over 500 stochastic reconstructions for multiregime models). Overall, we found little support of multiple phenotypic optima or differing rates of phenotypic evolution according to trophic strategy (general diet, OU5_diet_ and if it includes nectar or not, OU2_nectar_) for the four analyzed traits. Model fitting for PCa1, body size, and HWI identified a kappa model as the best model suggesting a punctuated mode of evolution. A multi-rate BM model was identified as the best model for the evolution of beak morphology (PCb1). The Fokker–Planck–Kolmogorov (FPK) model also received substantial support and was consistently ranked as the second-best model. Models that fit different optima to the different states were not among the best-fit models. This result was supported when using phylogenetic ANOVAs to test for differences in morphological traits among regimes (all *p*-values > 0.05). Early-burst (EB) models received consistently low statistical support, in agreement with the apparent rarity of this mode of evolution [34], which suggests that the process of psittaculid disparification bears little resemblance to classical adaptive radiation. When fitting a delta model, we found δ values > 1 in all cases. This result agrees with the results of disparity tests and suggests the existence of a trend towards a late-burst pattern of evolution for all analyzed traits, mostly for PCb1, whose δ value was at the default maximum, 3.

**Table 1:** Comparisons of different evolutionary model fits and parameters for the four phenotypic traits: PCa1, PCb1, body size, and HWI

Trait	Model	Loglik	AIC_c_	$\Delta$ AIC	Parameter
*PCa1*	1.BM	− 161.49	327.09	17.66	
	1.1.1 BMS *nectar*	− 161.35	328.91	19.48	
	1.1.2 BMS *diet*	− 156.56	325.88	16.45	
	1.2 delta	− 159.31	324.83	15.40	$\delta$ = 2.18
	**1.3 kappa**	**− 151.61**	**309.43**	**0**	$\kappa$= 0.39
	2. OU	− 158.97	324.15	14.72	$\alpha$= 0.026
	2.1.1 OU2 *nectar*	− 158.96	326.28	16.85	
	2.1.2 OU5 *diet*	− 157.75	330.53	21.10	
	3. FPK	− 157.61	323.58	14.15	
	4. EB	− 161.49	329.19	19.76	
	NIG*	− 207.06	422.48		
	JN*	− 215.06	438.48		
	JEM*	**− 147.95**	**303.99**		λ = 0.012, α = 28.8
*PCb1*	1. BM	− 223.62	451.35	24.09	
	1.1.1 BMS *nectar*	− 220.77	447.75	20.49	
	**1.2.1 BMS *diet***	**− 207.25**	**427.26**	**0**	
	1.2 delta	− 219.31	444.83	17.57	$\delta$= 2.99
	1.3 kappa	− 218.56	443.33	16.07	$\kappa$= 0.47
	2. OU	− 219.71	445.63	18.37	$\alpha$= 0.037
	2.1.1 OU2 *nectar*	− 219.30	446.96	19.70	
	2.1.2 OU5 *diet*	− 218.24	451.51	24.25	
	3. FPK	− 213.83	436.02	8.76	
	4. EB	− 223.62	453.45	26.19	
	NIG*	− 207.06	422.48		
	JN*	− 215.06	438.48		
	JEM*	**− 192.58**	**393.52**		λ = 0.008, α = 100.0
*Body size*	1. BM	96.44	− 188.77	10.94	
	1.1.1 BMS *nectar*	97.33	− 188.45	11.26	
	1.1.2 BMS *diet*	100.94	− 189.12	10.59	
	1.2 delta	96.55	− 186.89	12.82	$\delta$= 1.18
	**1.3 kappa**	**102.96**	**− 199.71**	**0**	$\kappa$= 0.43
	2. OU	96.90	− 187.59	12.12	$\alpha$= 0.005
	2.1.1 OU2 *nectar*	97.11	− 185.86	13.85	
	2.1.2 OU5 *diet*	96.60	− 178.17	21.54	
	3. FPK	100.72	− 193.08	6.63	
	4. EB	96.44	− 186.67	13.04	
	NIG*	**134.03**	**− 259.70**		
	JN*	89.47	− 170.58		
	JEM*	101.71	− 195.06		λ = 0.004, α = 38.8
*HWI*	1.BM	198.43	− 392.88	6.29	
	1.1.1 BMS *nectar*	199.45	− 392.69	6.48	
	1.1.2 BMS *diet*	202.77	− 392.78	6.39	
	1.2 delta	199.46	− 392.71	6.46	$\delta$ = 1.72
	**1.3 kappa**	**202.69**	**− 399.17**	**0**	$\kappa$= 0.60
	2. OU	199.69	− 393.17	6.00	$\alpha$= 0.017
	2.1.1 OU2 *nectar*	201.07	− 393.78	5.39	
	2.1.2 OU5 *diet*	204.19	− 393.35	5.82	
	3. FPK	201.88	− 395.40	3.77	
	4. EB	198.44	− 390.67	8.50	
	NIG*	195.20	− 382.04		
	JN*	198.01	− 387.66		
	JEM*	**207.96**	**− 407.56**		λ = 0.011, α = 26.8

Bolded rows represent the best-fit model for each class

We tested three main “classic” Gaussian models (Brownian Motion, BM; Ornstein–Uhlenbeck, OU; and Early-Burst, EB) and modifications of such models (BMS; OU2; OU5), Under these evolutionary models, the adaptive optimum of a lineage may wander gradually and freely (incremental change: BM), it may change gradually but remain stationary (incremental stationarity: OU), or it may change most rapidly following the initial diversification of a clade while decelerating over time (explosive change: EB). In addition, we tested a recently developed model (FPK), which assumes that characters evolve under both random diffusion and deterministic forces of any possible shape and strength. While in multi-OU model different peaks can only be discovered by certain lineages (which are inferred to be under the same regime), the FPK model does not impose such restriction. To test for pulsed evolution, three Lévy processes were fitted: a normally distributed jump process (JN) model, a variant of this model that implements an Expectation–Maximization approach (JEM), and a pure-normal inverse Gaussian process (NIG). Values are means from model fitting across 500 reconstructions of nectarivory/diet for models with multiple selective regimes (BMS nectar, BMS diet, OU2, and OU5). Mean Akaike information criterion (AIC_c_) is the averaged AIC_c_, and $\Delta$ AIC_c_ is the model’s mean AIC_c_ minus the minimum AIC_c_ between models. Bolded rows represent the best-fit model for each class (Lévy and non-Lévy processes) as indicated by the lowest AIC_c_ score. The phylogenetic parameters kappa ($\kappa$) and delta ($\delta$) and its associated models were also assessed in order to determine different processes (gradual *vs*. punctuated evolution; early *vs*. late evolution). κ = 1 indicates gradual evolution and κ = 0 indicates punctuated evolution. δ < 1 indicate temporally early trait evolution or ‘early-burst’, indicative of adaptive radiation whereas δ > 1 indicate temporally latter trait evolution, indicative of species‐specific adaptation (see main text for more details). Asterisks indicate jump (pulsed) models

**Fig. 4:**
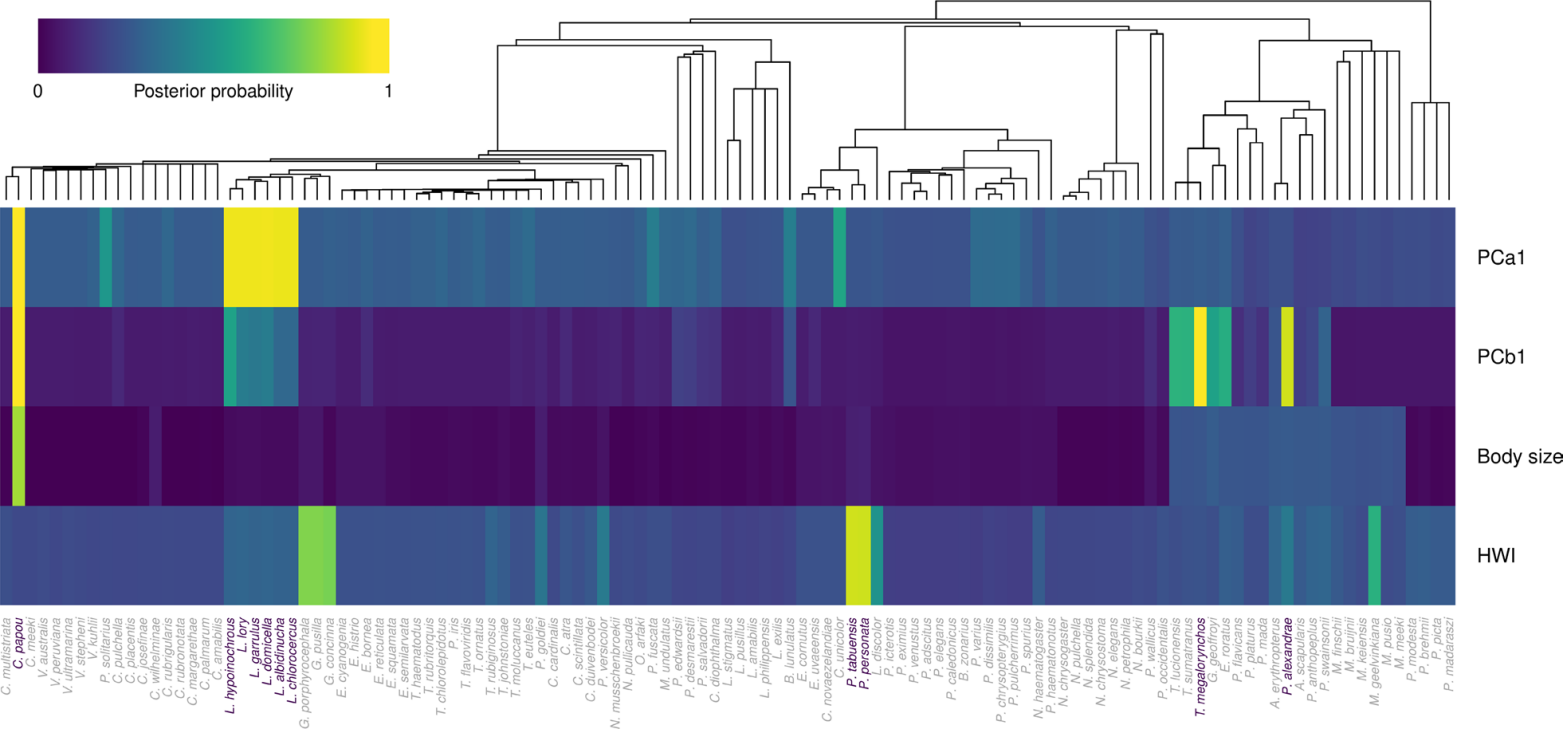
Heatmap illustrating the posterior probability of evolutionary jumps from the root to the terminal taxa for each trait following as estimated by ‘levolution’. The phylogeny used as basis is shown on the top. Species with posterior probabilities > 0.9 are highlighted. Estimates per branch are given in Additional file 1: Fig. S1.

**Fig. 5:**
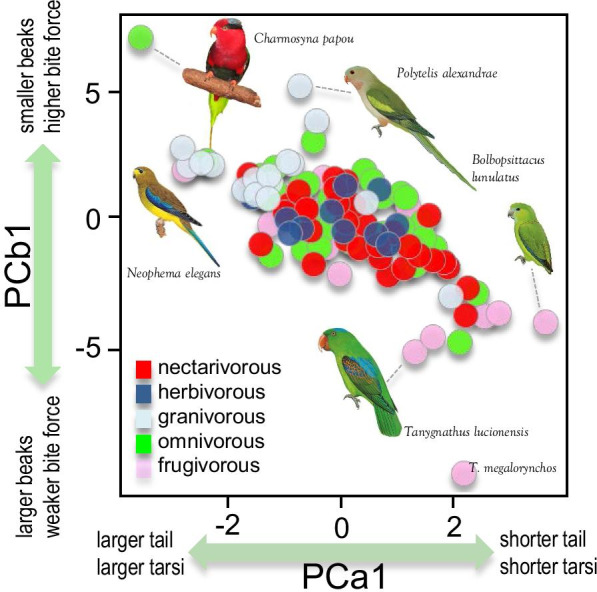
Two-dimensional morphospace plot delimited by PCa1 and PCb1. Psittaculid species are color-coded by dietary regime as in Fig. 1. Bird illustrations come from the Handbook of Birds of the World [84] and are reproduced with permission from Lynx Editions

Further, we found that a Lévy process model characterized by a period of stasis and pulses of rapid change provided a good fit for three of the four traits. Specifically, the most favored model for PCa1, PCb1, and HWI was a jump process model with Expectation–Maximization algorithm (JEM). This model indicates that phenotypic changes occur abruptly preceded and followed by longer periods of relative evolutionary stability, which agrees with the high support that the kappa (speciational) model received within the class of non-Lévy processes. According to the JEM model, jumps occurred the most frequently for PCa1 (λ = 0.012 per million years) and HWI (λ = 0.011) and a bit less often for PCb1 (λ = 0.008). Jumps were inferred to be of considerable strength with effect sizes (1/α) comparable to continuous evolution for about 25 (PCa1 and HWI) to 100 (PCb1) million years. An empirical Bayes estimate of the jump locations indicated that the jumps with the strongest support were located on the Loriini tribe (Fig. 4; Additional file 1: Fig. S3). These jumps corresponded to species occupying extreme positions of the morphospace (e.g., *Charmosyna papou*, *Tanygnathus megalorynchos*), which illustrates the lack of phenotypic differentiation among lineages belonging to different dietary regimes (Fig. 5).

The evolution of body size was best explained by the NIG model, which represents constant phenotypic change (i.e., continuous jumps). It agrees with the high support observed for the FPK model.

### Phenotypic diversification rates

**Fig. 6:**
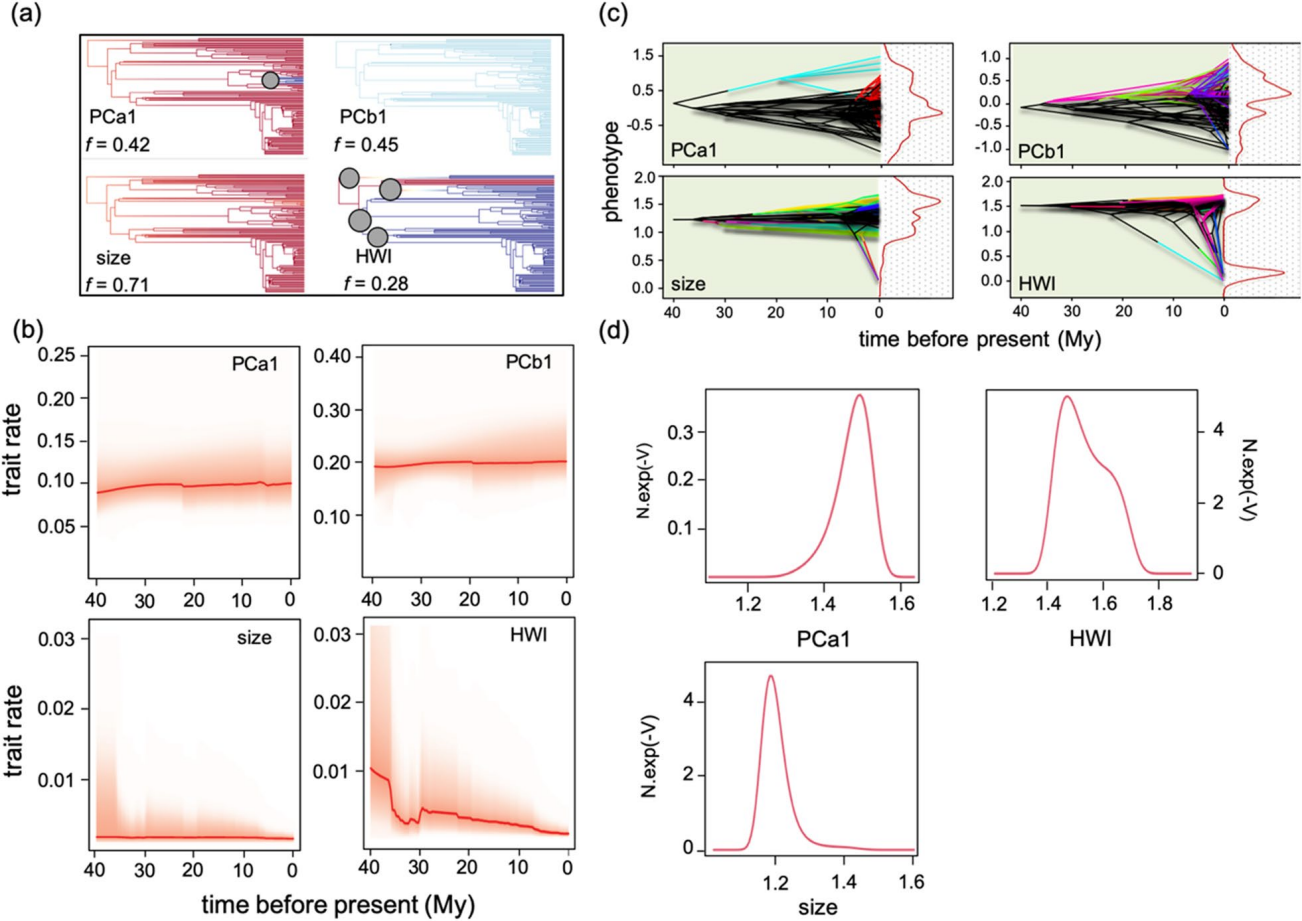
**a** Phylorate plots showing the most probable macroevolutionary rate shift configurations (and their frequencies, *f*) for the evolution of each trait across the phylogeny. Branch colors denote instantaneous rates (cold colors = slow, warm = fast). Circles denote the location of shifts in phenotypic rates. **b** Rate-through-time plots for phenotypic evolution rate (with 95% CI indicated by shaded areas) obtained using Bayesian analysis of macroevolutionary mixtures. **c** Phenograms from the ‘bayou’ analyses showing the distribution of each trait against the phylogeny. The curve in red (right panel) shows the distribution of the optimum/optima among lineages. **d** Macroevolutionary landscapes for those traits in which the FPK model was the best model or received high statistical support estimated using ‘BBMV’ [53] (see main text for details)

We investigated rates of continuous evolution in PCa1, PCb1, body size, and HWI, using the data-driven BAMM platform with no a priori designation of trophic regimes. With the exception of dispersal capacity (HWI), there was little collective posterior support for among-clade heterogeneity in phenotypic rates for all traits. No particular model with a specific shift time occurred at high frequency, suggesting that rate shifts could not be reduced to a single event at a strictly specified time (Fig. 6a). The most frequently supported model for PCb1 and body size included no internal rate shifts, whereas for PCa1 the model with the highest support included a single shift in the rate of diversification at the node giving rise to the rosellas *Platycercus* (*f* = 0.42) (Fig. 6a). Accordingly, rate-through-time plots for PCa1, PCb1, and body size revealed an almost constant process of phenotypic diversification with a subtle increase for the two first traits (Fig. 6b). We obtained similar results when restricting these analyses to the lories (Loriinae); only PCb1 showed marked variability over time (see Additional file 1: Figs. S4, S5).

*bayou* detected the existence of a unique selective regime for PCa1, PCb1, and body size, whereas HWI showed a bimodal pattern (Fig. 6c). These results agree with the macroevolutionary landscapes inferred by the FPK model (Fig. 6c).

## Discussion

Simpson envisaged a conceptual model of adaptive radiation in which lineages diversify into “adaptive zones” within a macroevolutionary adaptive landscape ([15]; see also [16]). For example, the diversification of feeding strategies has been accompanied by the diversification of skull and mandible shape in phyllostomid bats [35], and head and oral jaw morphology in Neotropical cichlids [36]. Here, we tested whether psittaculid (Australasian) parrots have evolved in a macro-evolutionary landscape where similar adaptive peaks in morphology exist for independently evolved taxa that occupy similar axes of trophic niche space. We found that lineages associated with different trophic regimes did not occupy clearly differentiated regions of the morphospace. Our results suggest that traits may evolve particularly fast around speciation or splitting events with no clear direction in this group (at least with regard to the analyzed variables). Thus, random walks rather than adaptive walks best describe how phenotypes evolve in Australasian parrots.

### Did the evolution of nectarivory trigger rapid lineage diversification in Australasian parrots?

According to our expectations for the psittaculid parrot radiation, nectarivory allowed an expansion into a new adaptive zone and subsequent diversification was essentially non-adaptive and might have been linked to repeated geoclimatic events [27]. Although our BAMM analysis detected a diversification-rate shift in Loriinae, which are mainly nectarivorous, this also occurred in Platycercinae, which are fundamentally omnivores or frugivores. This indicates that nectarivory might not have acted as a differential factor and fueled cladogenesis in the former radiation. Alternatively, this result may be due to the existence of different ecological opportunities apart from the shift to nectarivory, e.g., the expansion of open forests and more arid environments in mainland Australia. That is, different independent events could have led to initial increased rates of lineage diversification.

In line with the diversification analysis, disparity-through-time (DTT) results revealed a limited degree of trait disparity between subclades throughout the evolutionary history of this group, a pattern inconsistent with the idea of an ‘early-burst’ scenario [37]. For most traits, DTT plots showed that the observed disparity between subclades did not differ from that expected by chance (relative to the whole clade) during the first three-quarters of psittaculid history. However, this period of limited diversification is followed by a moderate change in the diversification dynamics of traits during the late Miocene-early Pliocene (around 6–5 Mya). This late pulse of diversification was especially notable in terms of beak morphology (PCb1) (Fig. 3c). This substantial increase in relative trait disparity over the last ~ 6 My coincides with a period of aridification and expansion of open forests in mainland Australia and the uplift of the frontal part of the present-day Papuan Fold Belt [38]. Thus, the onset of arid conditions coupled with orogenic processes may have spurred the diversification of this group [39]. In agreement with trends observed in the DTT analyses, delta values obtained by fitting the Pagel’s time-dependent model of trait evolution—especially those obtained for PCa1 and PCb1—support this view; phenotypic evolution was concentrated nearer the tips of the tree (see Table 1). These results contrast with those reported by [40] in Neotropical parrots (Arini; Psittacidae), which showed a concentration of size evolution and partitioning of size niches in its early history, once they colonized South America and coinciding with the first peak of the Andean orogenesis.

### Functional adaptations to nectarivory and other trophic strategies

Evolutionary model fitting revealed that models that fit different optima to the different regimes were not among the top-ranked ones. The kappa model was the best-fit for three of the four analyzed traits. The idea that trait evolution in this group follows a punctuated mode was further supported by the good fit of the jump model (JEM) within the Lévy class. Consequently, our findings suggest that a moderate amount of psittaculid morphological variance may have arisen through quantum leaps of trait variation representing excursions of lineages across trait space, which could lead to the founding of new clades with distinct morphology from their ancestors, and not from a gradual process ([3, 15]; see also [32]). The moderate support observed consistently for the non-uniform Fokker–Planck–Kolmogorov model (FPK) indicates that species’ traits in this clade may drift freely over time in phenotypic space (i.e., not restricted to certain regimes) but an ‘evolutionary potential’ seems to pull them towards different regions of morphospace (bounded evolution; [41]). That is, most psittaculid traits examined may have evolved under both random diffusion and a deterministic force. FPK is based on a constant-rate diffusion model and as such cannot model decelerating trait evolution or abrupt jumps in the value of the trait, as would be expected under quantum evolution, which limits the predictive power of this model [19]. It is likely that that best-fitting model would be one combining rare but rapid bursts of phenotypic change with bounded fluctuations on shorter timescales.

Specifically, regarding PCb1, we found no support for the idea that the evolution of different dietary strategies constitutes a selective factor of moderate importance in shaping beak morphology. It is probably due to the fact that other factors (i.e., changes in the genetic and selective background that are unrelated to diet) can counteract this selective factor and drive lineages through a random-walk process, with no clear direction. Our findings agree with that recently reported by [42] in a study on Psittaciformes including cockatoos. Bright and colleagues found that dietary preferences (summarized into two broad categories; high mechanically resistant food *vs*. low mechanically resistant food) accounted for a mere 2% of phenotypic variation in skull and beak shape [42]. In this sense, although diet and foraging behaviour have been traditionally invoked as significant predictors of cranial morphology in birds [43], recent studies both at broad and restricted phylogenetic scale have shown that the evolution of the avian skull is constrained by complex interactions among intrinsic and extrinsic factors. For instance, in a large-scale study, [22] reported low dimensionality and consistency of bill-shape variation within clades, and high dimensionality among clades. In the same vein, [44] concluded that, far from being an exemplary feeding adaptation, avian beak diversification may have been largely contingent on trade-offs and constraints, and [45] showed that trophic characters are significant but weak predictors of cranial form across large clades. At a smaller scale, [46] reported that the shapes of beaks in diurnal raptors are highly controlled by non-dietary factors—mainly allometry and phylogenetic inertia—whereas [47] observed a high degree of resemblance in terms of cranial morphology among meliphagid species (Australian honeyeaters) despite these having diverged in ecological space. Consequently, lineage-specific background effects and several constraints including phylogenetic legacy and the integration between beak and braincase shape may prevent lineages from adapting perfectly to the selective factor under study if the strength of selection is low [13, 48]. This fact may also explain why the evolution of this trait in particular is not characterized by large discrete jumps, but by small and continuous shifts. Noticeably, the obtained parameters indicate that the rate of beak shape evolution was faster in plant-feeding species (Additional file 1: Table S1). It agrees with the findings of [44], who reported that herbivory imposes high-performance demands on the beak as dietary transitions toward increased herbivory are correlated with evolutionary changes toward higher anterior mechanical advantages of the jaws. Hence, plant-feeding species like pygmy parrots (*Microsiptta*) species exhibit an exceptionally high biting force [44].

A kappa model was the most likely evolutionary model for body size. This in conjunction with the moderate support observed for the FPK model may indicate that diverse and multidirectional selective pressures are involved in the diversification process of this essential trait. The history of psittaculid body size seems to be too complex to be explained by a uniform process; body size correlates with virtually every aspect of species biology. Thus, due to its multitasking nature, it is subject to a tangled interplay among different constraints precluding the existence of uniform stabilizing selection and resulting in continuous jumps (i.e., small but constant phenotypic change) as indicated by the good fit observed for the normal inverse Gaussian (NIG) model. This echoes the outcome of other explorations of body size evolution in mammals and crocodylians [49, 50].

We also found low support for the directional or stabilizing selection models when analyzing the evolution of PCa1 despite the inferred optima for nectarivorous and non-nectarivorous were moderately different. This suggests that although the evolutionary dynamics for nectarivores on PCa1 were driven toward a significantly different phenotypic optimum, indicating larger tarsi and shorter tails, which could facilitate maneuverability when accessing and exploiting nectar resources, this dynamic was not at significantly different evolutionary rates relative to non-nectarivores. The distance between the observed species values and inferred optima suggests these lineages have been slow in their approaches to their adaptive peaks. Yet, these shifts seem to occur abruptly in a rapid manner—although not in a single direction—as revealed by the fit provided by the two different classes of models that support a punctuated mode of evolution; the kappa model and the jump model without matrix inversion (JEM) (see also [51]). Hence, the diversification process of this trait may involve a long period of stasis broken up by jumps that could have taken place on islands as most of them were associated to species endemic to Pacific islands (e.g., *Cyanoramphus unicolor*, endemic to the Antipodes Islands; *Lorius chlorocercus* endemic to the Solomon Islands; *Lorius albidinucha* endemic to the New Ireland island; *Phigys solitarius* endemic to the islands of Fiji; and *Prosopeia personata* endemic to the island Viti Levu) (see Additional file 1: Fig. S1).

Lastly, the model-fitting approach also favored a kappa model (and to a lesser extent, an FPK model) for the hand-wing index (HWI), a proxy for dispersal capacity. We found that HWI, unlike the other traits, tends to exhibit a bimodal pattern (subtle according to the FPK model and conspicuous according to the ‘bayou’ analyses; see Fig. 6c, d), whose distinct regimes do not correspond to any of those defined a priori here. Our findings are not in agreement with the hypothesis that nectarivores exhibit a greater dispersal capacity than non-nectarivorous species. In this regard, we found that nectarivorous species are represented across the Australasian region; 47% of species inhabit small islands (Maluku Islands, Sulawesi, Solomon Islands, and remaining archipelagos), whereas Australia and New Guinea harbor 32 and 21% of species, respectively (Fig. 1). Thus, it seems that nectarivory does not constitute a key factor in explaining the colonization and further expansion of some clades across islands; in fact, almost half (46%) of insular species (excluding New Guinea) do not feed on nectar. In this sense, it is likely that the colonization of these islands occurred in multiple and repeated episodes, a hallmark of many radiations in this region (e.g., [6]). In addition, it should be noted that a high HWI could also be highly adaptive in arid-adapted Australian species. This could contribute to explain the lack of a straightforward difference between nectarivorous and non-nectarivorous parrots in terms of dispersal capacity.

Phylorate plots obtained using BAMM revealed that phenotypic diversification was relatively constant for three of the four traits. This lack of rate shifts does not support the hypothesis that the adaptation to nectarivory led to an increase in diversification rates as a result of ecological release [4]. At this point, it should be noted that BAMM is widely used for inferring rates of morphological evolution, yet this method does not capture phases of pulsed evolution, which might be more important than clade-wide rate shifts. In addition, we did not detect a signal of strong selection driving nectarivorous species to different adaptive peaks from non-nectarivorous species in any of the analyzed traits, which could be partly attributed to the lack of a tight plant-bird relationship. Parrots are generalized flower visitors and their ecological relationships with plants are not as specialized as those of hummingbirds [33]. Nectar contains low levels of amino acids, vitamins, and trace minerals and thus nectarivorous parrots rely on other food sources (insects, pollen, manna, honeydew, and fruits) to meet their nutritional requirements [26]. Lastly, we must point out that nectarivorous parrots show some conspicuous adaptations to effectively feed on nectar like a brush-tipped tongue (and possibly other physiological aspects; [27]), which were not addressed in the present study.

## Conclusion

Our results for Australasian parrots do not support the hypothesis that trophic differences shaped their adaptive landscapes, which may be due to different background effects (e.g., genetics) and external factors. For instance, the markedly allopatric distribution of psittaculid species, mainly in Australia, may have played a fundamental role in facilitating the retention of ancestral morphology given reduced ecological interference among species, potentially contributing to the evolutionary stability of traits across species within major clades [52]. Our results suggest that beak shape is the result of a complex scenario in which several functional, behavioral factors interact, reflecting the multitasking nature of this trait [44, 45]. Finally, this study shows that recently developed non-uniform macroevolutionary models [53–55] can explain evolutionary patterns better than their predecessors, which are restricted to two overly simplistic scenarios: drift (BM) and stabilizing selection (OU).

## Methods

### Study system and phylogenetic inference

The Psittaculidae comprise 175–180 species distributed across Africa and Asia through to Australasia and Oceania, and vary moderately in body mass, from about 15–30 g (pygmy parrots *Micropsitta* spp., budgerigar *Melopsittacus undulatus* and the blue lorikeet *Vini peruviana*) to over 500 g in the eclectus parrot *Eclectus roratus*. We restricted the present study to the Australasian region (Australia, New Zealand, New Guinea, and surrounding islands) which harbors the greatest proportion of taxonomic diversity (39 of 45 genera and 68% of species of Psittaculidae inhabit this region) and has been suggested as an ancestral area of origin of all extant parrots [30].

We inferred the phylogenetic relationships among species from the consensus tree assembled by [56]. It consists of a calibrated Matrix Representation with Parsimony (MRP) phylogenetic supertree including all 398 extant species of parrots (order Psittaciformes). Since parrots have a poor fossil record [57], the Burgio supertree relies on well-accepted external fossil calibrations points, which are described in detail in [30, 31, 40]. To account for phylogenetic uncertainty in some analyses, we also used a sample of 500 trees obtained from BirdTree (www.birdtree.org) based on the ‘stage 2 parrot’ backbone phylogeny. BirdTree combines relaxed clock molecular trees of well-supported avian clades with a fossil calibrated backbone with representatives from each clade [58].

### Morphological and ecological data

We first characterized the overall morphology of each of the 117 species from average measurements on body length (size), tarsus length, tail length, culmen length, and the Hand Wing Index (HWI). These measurements were obtained from the *Handbook of Australian, New Zealand and Antarctic birds* [59] and *Parrots of the World* [52], except for HWI, which were retrieved from [60]. These traits are inherently linked to ecological and behavioral characteristics such as foraging niche and diet preferences. For instance, tarsus length has a strong influence on the foraging mode and provisioning behaviour, whereas the length of the tail affects maneuverability and stability and thus, confers aerodynamic properties [61]. The HWI describes the wing aspect ratio and, consequently, has been frequently used as a proxy of dispersal ability and flight efficiency (e.g., [62]). Specifically, HWI represents the distance between the tip of the first secondary feather and the tip of the longest primary feather (Kipp’s distance) corrected for wing size [60]. The first four variables (body size, culmen length, tarsus length, and tail length) were strongly correlated with each other after log-transformation. To avoid this and remove the confounding effects of body size while accounting for phylogenetic relationships among species, we performed phylogenetic regressions in ‘phytools’ using the BM method to obtain the correlation structure [63]. The obtained residuals for the three remaining traits (i.e., size-corrected variables) were used as input in a principal component analysis (PCA) in order to reduce the dimensionality of our trait dataset [64]. The PCA yielded a principal component axis (PCa1) that accounted for 70% of the total variance. Tarsus and culmen length were positively loaded (0.52 and 0.61, respectively), whereas tail length showed the opposite trend (factor loading: − 0.59).

We then focused in more detail on the beak morphology. We took advantage of the global dataset of morphological measurements compiled by J. Tobias and collaborators from live-caught individuals and preserved museum skins (e.g., [65]). This dataset includes five traits describing variation in beak shape [(1) beak length measured from fore-edge of cere to tip along the culmen; (2) beak length measured from the tip to the anterior edge of the nares; (3) beak depth measured at the anterior nares; (4) beak width measured at the anterior nares; and (5) gape width measured at the join of the mandibles] from a sample of adult individuals from each species (average number of measured individuals per species: 5.4). Since sexual size dimorphism is absent or minor in this taxonomic group [52], measurements on males and females were pooled together when computing the average values for each species. From these five linear measurements, we conducted another PCA in a similar way to that previously indicated once these were log-transformed and size-corrected. The first axis (PCb1) captured the dominant proportion of variation in the beak dataset (89% of the total variance) All factor loadings were negative and > 0.40. Lower values of PCb1 indicate species with relatively larger and less downward inclined beaks, the great-billed parrot *Tanygnathus megalorynchos* being the species with the lowest score. Higher PCb1 values indicate species with relatively smaller and more curved beaks (and high bite force) like the Papuan lorikeet *Charmosyna papou* or the superb parrot *Polytelis swainsonii*. Species’ scores on these two morphological axes (PCa1—appendices—and PCb1—beak shape), body size and HWI values were used as input in subsequent analyses.

We defined five main trophic groups [(1) omnivore; (2) granivore; (3) frugivore; (4) herbivore; (5) nectarivore) according to dietary information obtained from the literature [56, 66, 67]. The diet of Australasian parrots is reasonably well documented and we found no discrepancies among sources. Subsequently, we defined a broader category in which we discerned between species that include nectar in their diet and those that do not (Fig. 1).

### Lineage diversification

We tested for heterogeneity in diversification rates across the phylogeny using Bayesian analysis of macroevolutionary mixtures (BAMM) [68, 69]. This program is oriented entirely towards detecting and quantifying heterogeneity in evolutionary rates, allowing to infer mixtures of time-dependent and clade-specific lineage or phenotypic rate regimes on phylogenetic trees. BAMM uses a reversible jump Markov chain Monte Carlo algorithm to explore the universe of candidate cladogenesis models. Shifts in evolutionary rates are detected automatically, with no a priori designations, and can occur at nodes or along branches. After computing tree-appropriate rate priors using the R package BAMMtools [70], we ran BAMM for 100 million generations on the MRP tree, sampling every 20,000 generations. To account for incomplete sampling, we specified the percentage of species that have been sampled by setting the ‘globalSamplingFraction’ parameter in our control file. We used the ‘coda’ package [71] to explore the Markov chain Monte Carlo (MCMC) output and check for convergence (50% generations as burn-in).

### Macroevolutionary modeling

- (1) *Disparity*

We analyzed the temporal dynamics of traits’ disparity using disparity-through-time (DTT) plots. This method computes the phenotypic disparity of each subclade relative to the phenotypic disparity of all taxa, and plots the change in average relative subclade disparity through time [72]. The observed DTT trajectory was compared to that expected according to pure Brownian motion (2500 simulations) and deviations were evaluated using the Morphological Disparity Index (MDI), which represents the area between the curves illustrating the observed and expected patterns of morphological divergence. Disparity values significantly greater than the null suggest that subclades overlap, and all contain a significant proportion of variation found throughout the entire group at a given time. Negative disparity indicates that morphological variation is partitioned among subclades, indicating that each subclade occupies a distinct region of the morphospace. In adaptive radiations, disparity is highly partitioned among clades early in the radiation, with each lineage holding little of the total disparity, and significant negative MDI values are expected. Relative subclade disparity is expected to decrease linearly towards the present if phenotypic evolution has occurred under a constant rate process. Trait disparity-through-time (DTT) analyses were conducted using the R package ‘geiger’ [72] and the rank envelope test developed by [73]. This test is employed to detect non-random bursts of trait evolution and investigate by standard significance testing if the empirical curve is found within the most extreme ranked curves. The rank envelope test computes a range of *p*‐values that encompasses the most liberal and most conservative *p*‐values, respectively, and shows better type I and II error rate than the original pointwise envelope test (see [73] for more details).

- (2) *Trait model fitting*

We then assessed several evolutionary models to find the best fit to explain the evolution of both overall morphology (body size, PCa1 and HWI) and beak morphology (PCb1) in Australasian parrots. Firstly, we fitted a single-rate Brownian motion (BM) model. This model assumes a random walk (time-homogeneous) process in which individual trait values fluctuate at random and increase uniformly as a function of time and thus, indicates undirected and unconstrained stochastic change [74]. That is, traits change as a result of genetic drift (neutral evolutionary change). The dynamics of this model are governed by a single parameter (σ^2^) that determines the rate at which independent lineages diffuse away from an ancestral condition. A non-uniform multi-regime BM model (BMS) that allows σ^2^ to differ among states, and where the parameter α is set to zero (diffusive model of evolution; [75]) and a model with decreasing rates of character evolution through time called ‘early-burst’ EB model (also known as ‘ACDC’ model) [34] were also fitted. We expected that the rate at which a trait changes through time (σ^2^) would be higher (i.e., faster evolution) in more specialized niches compared to broader generalist niches [76]. In addition, we incorporated two modifications of the uniform BM process that capture other conceivable ways in which traits might evolve. Specifically, a model (delta) with an added parameter ($\delta$) to model a rate shift indicating a late or early-burst fashion (i.e., whether trait evolution has accelerated or slowed down over time), and a model that evaluates whether species traits have evolved according to phyletic gradualisms or punctuated evolution through history (kappa model) [77].

Increasing in complexity, the Ornstein–Uhlenbeck (OU) process is a modification of BM that incorporates attraction (α) to a trait ‘optimum’ (θ). OU models describe the evolution of a particular trait towards or around a stationary peak or optimum value, at a given evolutionary rate (i.e., constrained morphological evolution) [14]. We also examined multipeak OU models in which these parameters vary with dietary regime allowing us to estimate adaptation and strength of selection towards separate phenotypic optima. We considered an OU model with attraction to five distinct selective optima corresponding to different dietary groups (OU5 = OU_diet_) and another two-regime model with different optima for nectarivorous and non-nectarivorous species (OU2 = OU_nectar_). In these models, different peaks can only be discovered by certain lineages (which are inferred to be under the same regime), and do not affect the evolutionary dynamics of other regions of the tree.

As an alternative, we employed the Fokker–Planck–Kolmogorov (FPK) model recently devised by [19], which can approximate adaptive landscapes with more complex shapes than those assumed by OU models, thereby accommodating scenarios of disruptive and directional selection. According to the FPK model, the trait under random diffusion and is also subject to an ‘evolutionary potential’ (*V*) that creates a force that pulls the trait towards specific regions of the trait interval [19]. That is, the trait evolves according to bounded random diffusion (or biased random-walk). Hence, while OU models with several optima might be better at describing transitions (i.e., situations in which a lineage shifts to another adaptive zone [15], multi-peak FPK might represent more genuine diversifying selection towards alternative phenotypic optima [19]. In other words, the OU model is specifically devised to model adaptation in the form of stabilizing selection around an optimal value [13]. In contrast, the FPK model and extended versions (Bounded Brownian Motion, BBMV) are useful to describe a scenario in which traits can drift freely over time in phenotypic space, except that they are confined between two bounds [41]. Discerning between both cases is of utmost importance since several studies have shown that neutral evolution between bounds produces patterns closely resembling the ones obtained under an OU process [78].

Finally, to assess the possible role of pulsed processes in trait evolution, we fitted Lévy process models. Specifically, a pure normal inverse Gaussian process (NIG), which captures constant rapid adaptation [79], and two evolutionary jumps processes that model stasis followed by sudden shifts in trait values between adaptive zones. The evolutionary jumps models were implemented following the approach described in [80], which assumes that jump effects are normally distributed (pure jump-normal model; JN), and a variant of this approach (JEM) that uses an alternative optimization that does not require matrix inversions to sample jump configurations (see [32] for further details). These pulsed models are intended to evaluate the punctuated component of evolutionary processes and were not assessed in conjunction with the conventional Gaussian models (and its modifications) described above (BM, EB, and OU), which depict evolutionary patterns (gradual change, gradual stasis, and adaptive radiation) that differ in form and mode (see e.g., [51]).

Model fitting was carried out using the packages ‘geiger’ [72], ‘mvMORPH’ [75], ‘BBMV’ [53] and ‘pulsR’ [79] in R 4.0.2, as well as with the software ‘levolution’ (https://bitbucket.org/wegmannlab/levolution, commit d9898f4, [32]). The relative fit of models was assessed using the Akaike’s Information Criterion corrected for small sample sizes (AICc) and the $\Delta$ AICc metric. To consider uncertainty in the ancestral dietary regime, all multiregime models were run on 500 stochastically mapped trees generated using stochastic character mapping (SIMMAP), an empirical Bayes procedure whereby character substitutions are mapped onto the nodes and branches of a phylogenetic tree. This MCMC approach whereby character substitutions are mapped onto the nodes and branches of a phylogenetic tree was implemented using the *make.simmap* function in ‘phytools’ [81]. The best-fit model of character evolution for the evolution of nectarivory (2-regimes) and diet (5-regimes) was determined by fitting an equal-rates (“ER”) model, a symmetric (“SYM”) model, an all-rates-different (“ARD”) model to the data set using the function *fitDiscrete* in ‘geiger’. The ER and the SYM transition-rate model were employed to estimate the ancestral states of nectarivory and diet, respectively, as these models yielded the lowest AICc [82]. Complementarily, we used simulation-based phylogenetic ANOVA [83] to test for differences in morphological traits among diet categories. We also used ‘OUwie’ [76] to estimate trait optima and rate of evolution for each regime.

### Phenotypic diversification rates

To estimate rates of evolution for continuous traits (body size, HWI, PCa1, and PCb1), we used BAMM in a similar way to that previously described. We performed these analyses across the entire phylogeny and for lories separately to determine whether Loriinae, the clade in which nectarivory is the most frequent feeding strategy, underwent an increase in diversification rate relative to other psittaculid lineages. Evolutionary rate dynamics were visualized from BAMM output using phylorate and rate-through-time plots generated in BAMMtools [70].

In addition, we explored the statistical patterns of the trait data using the reversible-jump algorithm implemented in ‘bayou’ [20]. ‘bayou’ employs Bayesian rjMCMC to identify regimes without a priori hypotheses in phylogenetic comparative data. This Bayesian approach alleviate many of the challenges inherent to fitting OU models, due to ridges in the likelihood space. By examining the posterior distribution, rather than point estimates, this method provides a more realistic interpretation of evolutionary models. Convergence of optima in ‘bayou’ was assessed by visual comparison of data within the phenogram.

## Supplementary Information

**Additional file 1: Table S1.** Trait optima (θ) and rate of evolution or ‘drift variance’ (σ^2^) estimated for both categorizations (*nectarivory*, 2-regimes: N = nectarivorous, NN = non-nectarivorous; *diet*, 5-regimes: O = omnivory; F = fruits; P = plants; S = seeds; N = nectar) using the full model (OUMVA) in ‘OUwie’ (Beaulieu & O’Meara, 2015). This model all allows all parameters (θ, σ^2^ and α) to vary by regime. Due to its complexity the likelihood of the OUMVA model for both PCa1 and size did not converge when discerning among the five dietary regimes. It resulted in biologically unfeasible θ values and negative eigenvalues of the Hessian matrix so instead, we reported values obtained using a simpler model (OUMA), which assumes distinct θ and α for each regime while keeping constant σ^2^. **Fig. S1.** Stochastically mapped discrete character history of diet in psittaculid parrots overlaid on a traitgram plot of PCa1, PCb1, body size, and HWI. The posterior probabilities from stochastic mapping are represented at each node. **Fig. S2.** Stochastically mapped discrete character history of nectarivory in psittaculid parrots overlaid on a traitgram plot of PCa1, PCb1, body size, and HWI. The posterior probabilities from stochastic mapping are represented at each node. **Fig. S3.** Posterior probability that a branch has a jump (left) and the posterior mean number of jumps per branch (right) on a color scale from black (no jump) to red (jumps) for each trait (*a*: body size, *b*: PCa1; *c*: PCb1; and *d*: HWI) following the approach devised by [32]. **Fig. S4.** Rate-through-time plots for phenotypic evolution rate (with 95% confidence intervals indicated by shaded areas) in lories (Loriinae) obtained using Bayesian analysis of macroevolutionary mixtures. **Fig. S5.** Rates of phenotypic evolution, estimated using BAMM, on different traits (a: PCa1, b: PCb1, c: body size, and d: HWI) during the radiation of Australasian psittaculid parrots. Smaller multipanel phylogenies (a-d) show the distinct rate-shift configurations with the highest posterior probability. For each distinct shift configuration, the locations of rate shifts are shown as circles, with circle size proportional to the marginal probability of the shift. Text labels (e.g., *f* = 0.25) indicate the posterior probability of each shift configuration.

## Data Availability

The datasets used in the current study are available from the corresponding author on reasonable request.

## References

[1] Losos JB, Glor RE, Kolbe JJ, Nicholson K (2006). Adaptation, speciation, and convergence: a hierarchical analysis of adaptive radiation in Caribbean *Anolis* lizards. Ann Mo Bot Gard.

[2] Martin CH, Feinstein LC (2014). Novel trophic niches drive variable progress towards ecological speciation within an adaptive radiation of pupfishes. Mol Ecol.

[3] Simpson GG (1953). The major features of evolution.

[4] Yoder JB, Clancey E, des RochesEastman SJM, Gentry L (2010). Ecological opportunity and the origin of adaptive radiations. J Evol Biol.

[5] Stroud JT, Losos JB (2016). Ecological opportunity and adaptive radiation. Annu Rev Ecol Evol Syst.

[6] García-Navas V, Rodríguez-Rey M, Westerman M (2018). Bursts of morphological and lineage diversification in modern dasyurids, a “classic” adaptive radiation. Biol J Lin Soc.

[7] Brennan IG, Oliver PM (2017). Mass turnover and recovery dynamics of a diverse Australian continental radiation. Evolution.

[8] Schluter D (2000). The ecology of adaptive radiation.

[9] Claramunt S (2010). Discovering exceptional diversifications at continental scales: the case of the endemic families of Neotropical suboscine passerines. Evolution.

[10] Cadena CD, Cuervo AM, Céspedes LN, Bravo GA (2020). Systematics, biogeography, and diversification of *Scytalopus* tapaculos (Rhinocryptidae), an enigmatic radiation of Neotropical montane birds. Auk.

[11] Day JJ, Martins FC, Tobias JA, Murrell DJ (2020). Contrasting trajectories of morphological diversification on continents and islands in the Afrotropical white-eye radiation. J Biogeogr.

[12] Rundell RJ, Price TD (2009). Adaptive radiation, nonadaptive radiation, ecological speciation and nonecological speciation. Trends Ecol Evol.

[13] Hansen TF (1997). Stabilizing selection and the comparative analysis of adaptation. Evolution.

[14] Butler MA, King AA (2004). Phylogenetic comparative analysis: a modeling approach for adaptive evolution. Am Nat.

[15] Simpson GG (1944). Tempo and mode in evolution.

[16] Losos JB (2010). Adaptive radiation, ecological opportunity, and evolutionary determinism. Am Nat.

[17] Beaulieu JM, Jhwueng DC, Boettiger C, O’Meara BC (2012). Modeling stabilizing selection: expanding the Ornstein-Uhlenbeck model of adaptive evolution. Evolution.

[18] Ingram T, Mahler DL (2013). *SURFACE*: detecting convergent evolution from comparative data by fitting Ornstein-Uhlenbeck models with stepwise AIC. Methods Ecol Evol.

[19] Boucher FC, Démery V, Conti E, Harmon LJ, Uyeda J (2018). A general model for estimating macroevolutionary landscapes. Syst Biol.

[20] Uyeda JC, Harmon LJ (2014). A novel Bayesian method for inferring and interpreting the dynamics of adaptive landscapes from phylogenetic comparative data. Syst Biol.

[21] Davis A, Unmack P, Pusey BJ, Pearson RG, Morgan DL (2014). Evidence for a multi-peak adaptive landscape in the evolution of trophic morphology in terapontid fishes. Biol J Lin Soc.

[22] Cooney CR, Bright JA, Capp EJR, Chira AM, Hughes EC, Moody CJA, Nouri LO, Varley ZK, Thomas GH (2017). Mega-evolutionary dynamics of the adaptive radiation of birds. Nature.

[23] Reaney AM, Bouchenak-Khelladi Y, Tobias JA, Abzhanov A (2020). Ecological and morphological determinants of evolutionary diversification in Darwin's finches and their relatives. Ecol Evol.

[24] Jønsson KA, Fabre PH, Fritz SA, Etienne RS, Ricklefs RE, Jørgenson TB (2012). Ecological and evolutionar determinants for the adaptive radiation of the Madagascaran vangas. Proc Natl Acad Sci USA.

[25] Burin G, Kissling W, Guimarães P, Şekercioğlu CH, Quental TB (2016). Omnivory in birds is a macroevolutionary sink. Nat Commun.

[26] Gartrell BD (1980). The nutritional, morphologic, and physiologic bases of nectarivory in Australian birds. J Avian Med Surg.

[27] Schweizer M, Güntert M, Seehausen O, Leuenberger C, Herwtig ST (2014). Parallel adaptations to nectarivory in parrots, key innovations and the diversification of the Loriinae. Ecol Evol.

[28] Fleischer RC, James HF, Olson SL (2008). Convergent evolution of Hawaiian and Australo-Pacific honeyeaters from distant songbird ancestors. Curr Biol.

[29] Marki PZ, Kennedy JD, Cooney CR (2019). Adaptive radiation and the evolution of nectarivory in a large songbird clade. Evolution.

[30] Schweizer M, Seehausen O, Hertwig ST (2011). Macroevolutionary patterns in the diversification of parrots—effects of climate change, geological events and key innovations. J Biogeogr.

[31] Schweizer M, Wright TF, Peñalba JV, Schirtzinger EE, Joseph L (2015). Molecular phylogenetics suggests a New Guinean origin and frequent episodes of founder-event speciation in the nectarivorous lories and lorikeets (Aves: Psittaciformes). Mol Phylogenet Evol.

[32] Duchen P, Leuenberger C, Sándor M, Szilágyi SM, Harmon LJ, Eastman J, Schweizer M, Wegmann D (2017). Inference of evolutionary jumps in large phylogenies using Lévy processes. Syst Biol.

[33] Fleming TH, Muchhala N (2008). Nectar-feeding bird and bat niches in two worlds: pantropical comparisons of vertebrate pollination systems. J Biogeogr.

[34] Harmon LJ (2010). Early bursts of body size and shape evolution are rare in comparative data. Evolution.

[35] Monteiro LR, Nogueira MR (2011). Evolutionary patterns and processes in the radiation of phyllostomid bats. BMC Evol Biol.

[36] Arbour JH, López-Fernández H (2014). Adaptive landscape and functional diversity of Neotropical cichlids: implications for the ecology and evolution of Cichlinae (Cichlidae; Cichliformes). J Evol Biol.

[37] Harmon LJ, Schulte PM, Larson A, Losos JB (2003). Tempo and mode of evolutionary radiation in Iguanian lizards. Science.

[38] Haig DW, Medd D (1996). Latest Miocene to Early Pliocene bathymetric cycles related to tectonism, Puri Anticline, Papuan Basin, Papua New Guinea. Aust J Earth Sci.

[39] Schweizer M, Güntert M, Hertwig ST (2013). Out of the Bassian province: historical biogeography of the Australasian platycercine parrots (Aves, Psittaciformes). Zoolog Scr.

[40] Schweizer M, Hertwig ST, Seehausen O (2014). Diversity versus disparity and the role of ecological opportunity in a continental bird radiation. J Biogeogr.

[41] Boucher FC, Démery V (2016). Inferring bounded evolution in phenotypic characters from phylogenetic comparative data. Syst Biol.

[42] Bright JA, Marugán-Lobón J, Rayfield EJ, Cobb SN (2019). The multifactorial nature of beak and skull shape evolution in parrots and cockatoos (Psittaciformes). BMC Evol Biol.

[43] Gill FB (1995). Ornithology.

[44] Navalón G, Bright JA, Marugán-Lobón J, Rayfield EJ (2019). The evolutionary relationship among beak shape, mechanical advantage, and feeding ecology in modern birds. Evolution.

[45] Felice RN, Tobias JA, Pigot AL, Goswami A (2019). Dietary niche and the evolution of cranial morphology in birds. Proc R Soc B Biol Sci.

[46] Bright JA, Marugán-Lobón J, Cobb SN, Rayfield EJ (2016). The shapes of bird beaks are highly controlled by nondietary factors. Proc Natl Acad Sci USA.

[47] Miller ET, Wagner SK, Harmon LJ, Ricklefs RE (2017). Radiating despite a lack of character: ecological divergence among closely related, morphologically similar honeyeaters (Aves: Meliphagidae) co-occurring in arid Australian environments. Am Nat.

[48] Navalón G, Marugán-Lobón J, Bright JA, Cooney CR, Rayfield EJ (2020). The consequences of craniofacial integration for the adaptive radiations of Darwin’s finches and Hawaiian honeycreepers. Nat Ecol Evol.

[49] Price SA, Hopkins SSB (2015). The macroevolutionary relationship between diet and body mass across mammals. Biol J Lin Soc.

[50] Godoy PL, Benson RBJ, Bronzati M (2019). The multi-peak adaptive landscape of crocodylomorph body size evolution. BMC Evol Biol.

[51] Powell S, Price SL, Kronauer DJC (2020). Trait evolution is reversible, repeatable, and decoupled in the soldier castle of turtle ants. Proc Natl Acad Sci USA.

[52] Forshaw JM. Parrots of the world: a field guide. Helm. 2010. 336 pp.

[53] Boucher FC (2019). *BBMV*: an R package for the estimation of macroevolutionary landscapes. Ecography.

[54] Harmon LJ. *Phylogenetic comparative methods learning from trees*. Book version 1.4, released on 15th March 2019 under CC-BY-4.0 license. 2019. https://lukejharmon.github.io/pcm/chapters/.

[55] Blomberg SP, Rathnayake SI, Moreau CM (2020). Beyond Brownian Motion and the Ornstein-Uhlenbeck process: stochastic diffusion models for the evolution of quantitative characters. Am Nat.

[56] Burgio KR, Davis KE, Dreiss LM, Klingbeil BT, Cisneros LM (2019). Phylogenetic supertree and functional trait database for all extant parrots. Data Brief.

[57] Mayr G (2009). Paleogene fossil birds.

[58] Jetz W, Thomas GH, Joy JB, Hartmann AO, Mooers AO (2012). The global diversity of birds in space and time. Nature.

[59] Higgins PJ (Ed.) Handbook of Australian, New Zealand and Antarctic birds. Volume 4: Parrots to Dollarbird. Oxford University Press, Melbourne; 1999.

[60] Sheard C, Neate-Clegg MHC, Alioravainen N, Jones SEI, Vincent C (2020). Ecological drivers of global gradients in avian dispersal inferred from wing morphology. Nat Commun.

[61] Fitzpatrick S (1987). Patterns of morphometric variation in birds’ tails: length, shape and variability. Biol J Lin Soc.

[62] Kennedy JD (2016). The influence of wing morphology upon the dispersal, geographical distributions and diversification of the Corvides (Aves; Passeriformes). Proc R Soc Lond B.

[63] Revell LJ (2009). Size-correction and principal components for inter-specific comparative studies. Evolution.

[64] Polly PD, Lawing AM, Fabre A-C, Goswani A (2013). Phylogenetic principal components analysis and geometric morphometrics. Hystrix Ital J Mammal.

[65] Pigot AL, Sheard C, Miller ET, Bregman T, Freeman B (2020). Macroevolutionary convergence connects morphological form to ecological function in birds. Nat Ecol Evol.

[66] Juniper T, Parr M (1998). Parrots: a guide to the parrots of the world.

[67] Wilman H, Belmaker J, Simpson J, de la Rosa C (2014). EltonTraits 1.0: species-level foraging attributes of the world’s birds and mammals. Ecology.

[68] Rabosky DL (2006). Likelihood methods for inferring temporal shifts in diversification rates. Evolution.

[69] Rabosky DL (2014). Automatic detection of key innovations, rate shifts, and diversity-dependence on phylogenetic trees. PLoS ONE.

[70] Rabosky DL, Grundler M, Anderson C, Title P (2014). *BAMMtools*: an R package for the analysis of evolutionary dynamics on phylogenetic trees. Methods Ecol Evol.

[71] Plummer M, Best N, Cowles K, Vines K (2006). CODA: convergence diagnosis and output analysis for MCMC. R News.

[72] Pennell MW, Eastman JM, Slater GJ, Brown JW, Uyeda JC, FitzJohn RG, Alfaro ME, Harmon LJ (2014). Geiger v2.0: an expanded suite of methods for fitting macroevolutionary models to phylogenetic trees. Bioinformatics.

[73] Murrell DJ (2018). A global envelope test to detect non-random bursts of trait evolution. Methods Ecol Evol.

[74] Felsenstein J (1985). Phylogenies and the comparative method. Am Nat.

[75] Clavel J, Escarguel G, Merceron G (2015). mvMORPH: an R package for fitting multivariate evolutionary models to morphometric data. Methods Ecol Evol.

[76] Beaulieu JM, O’Meara BC. *OUwie*: analysis of evolutionary rates in an OU framework. R package version 1.45. 2015. http://cran.rproject.org/web/packages/OUwie/index.html.

[77] Pagel M (1999). Inferring the historical patterns of biological evolution. Nature.

[78] Revell LJ, Harmon LJ, Collar DC (2008). Phylogenetic signal, evolutionary process, and rate. Syst Biol.

[79] Landis MJ, Schraiber JG (2017). Pulsed evolution shaped modern vertebrate body sizes. Proc Natl Acad Sci USA.

[80] Landis MJ, Schraiber JG, Liang M (2013). Phylogenetic analysis using lévy processes: finding jumps in the evolution of continuous traits. Syst Biol.

[81] Revell LJ (2012). *phytools*: an R package for phylogenetic comparative biology (and other things). Methods Ecol Evol.

[82] Burnham KP, Anderson DR (2002). Model selection and multimodel inference: a practical information-theoretical approach.

[83] Garland T, Dickerman AW, Janis CM, Jones JA (1993). Phylogenetic analysis of covariance by computer simulation. Syst Biol.

[84] Collar NJ, del Hoyo J, Elliott A, Sargatal J (1998). Family Psittacidae (parrots). Handbook of the birds of the world.

