# Supplementary material for: Trophic niche shifts and phenotypic trait evolution are largely decoupled in Australasian parrots
Source: BMC Ecol Evol. 2021 Nov 27;21:212. doi: 10.1186/s12862-021-01940-4 (PMC8626917; doi:10.1186/s12862-021-01940-4)

**Supplementary material**

**Trophic niche shifts and phenotypic trait evolution are largely decoupled in Australasian parrots**

Vicente García-Navas^1,2,3^, Joseph A. Tobias^4^, Manuel Schweizer^5^, Daniel Wegmann^6,7^, Richard Schodde^8^, Janette A. Norman^9^ and Les Christidis^9^

^1^ *Department of Integrative Ecology, Doñana Biological Station EBD (CSIC), Seville, Spain*

^2^ *Department of Evolutionary Biology and Environmental Studies, University of Zurich, Zurich, Switzerland*

^3^ *Centre for Ecology, Evolution and Environmental Changes (cE3c), University of Lisbon, Lisbon, Portugal*

^4^ *Department of Life Sciences (Silwood Park), Faculty of Natural Sciences, Imperial College London, London, UK*

^5^ *Natural History Museum of Bern, Bern, Switzerland*

^6^ *Department of Biology, University of Fribourg, Fribourg, Switzerland*

*^7^ Swiss Institute of Bioinformatics, Fribourg, Switzerland*

^8^ *Australian National Wildlife Collection, CSIRO Sustainable Ecosystems, Canberra, Australia*

^9^ *Southern Cross University, Coffs Harbour, NSW, Australia*

### **Corresponding author**

Correspondence to Vicente García-Navas:

vicente.garcianavas@ebd.csic.es

**Table S1.** Trait optima (θ) and rate of evolution or ‘drift variance’ (σ^2^) estimated for both categorizations (*nectarivory*, 2-regimes: N = nectarivorous, NN = non-nectarivorous; *diet*, 5-regimes: O = omnivory; F = fruits; P = plants; S = seeds; N = nectar) using the full model (OUMVA) in ‘OUwie’ (Beaulieu & O’Meara, 2015). This model all allows all parameters (θ, σ^2^ and α) to vary by regime. Due to its complexity the likelihood of the OUMVA model for both PCa1 and size did not converge when discerning among the five dietary regimes. It resulted in biologically unfeasible θ values and negative eigenvalues of the Hessian matrix so instead, we reported values obtained using a simpler model (OUMA), which assumes distinct θ and α for each regime while keeping constant σ^2^.

|  | PCa1 | | PCb1 | | size | | HWI | |
| --- | --- | --- | --- | --- | --- | --- | --- | --- |
|  | θ | σ^2^ | θ | σ^2^ | θ | σ^2^ | θ | σ^2^ |
| *nectar* |  | | | | | | | |
| N | -0.22 | 0.03 | -0.62 | 0.43 | 0.48 | 1.68^e-3^ | 1.63 | 0.01 |
| NN | 2.25 | 0.02 | -0.18 | 0.45 | 1.31 | 7.89^e-4^ | 1.58 | 0.01 |
| *diet* |  | | | | | | | |
| O | 1.77 | 0.05 | -0.61 | 0.06 | 1.45 | 1.1^e-3^ | -0.34 | 4.8^e-4^ |
| F | 19.17 | 0.06 | -2.67 | 0.07 | 1.37 | 5.7^e-3^ | 1.60 | 2.8^e-6^ |
| P | -0.24 | 0.02 | -1.09 | 9.0^e-3^ | -2.99 | 3.3.^e-5^ | 1.37 | 3.5^e-4^ |
| S | -0.54 | 0.10 | 7.93 | 0.03 | 1.28 | 4.1^e-3^ | 1.64 | 8.7^e-5^ |
| N | 1.36 | 0.03 | -0.14 | 0.05 | 1.33 | 4.0^e-3^ | 1.63 | 1.0^e-4^ |

**Figure S1.** Stochastically mapped discrete character history of diet in psittaculid parrots overlaid on a traitgram plot of PCa1, PCb1, body size, and HWI. The posterior probabilities from stochastic mapping are represented at each node.


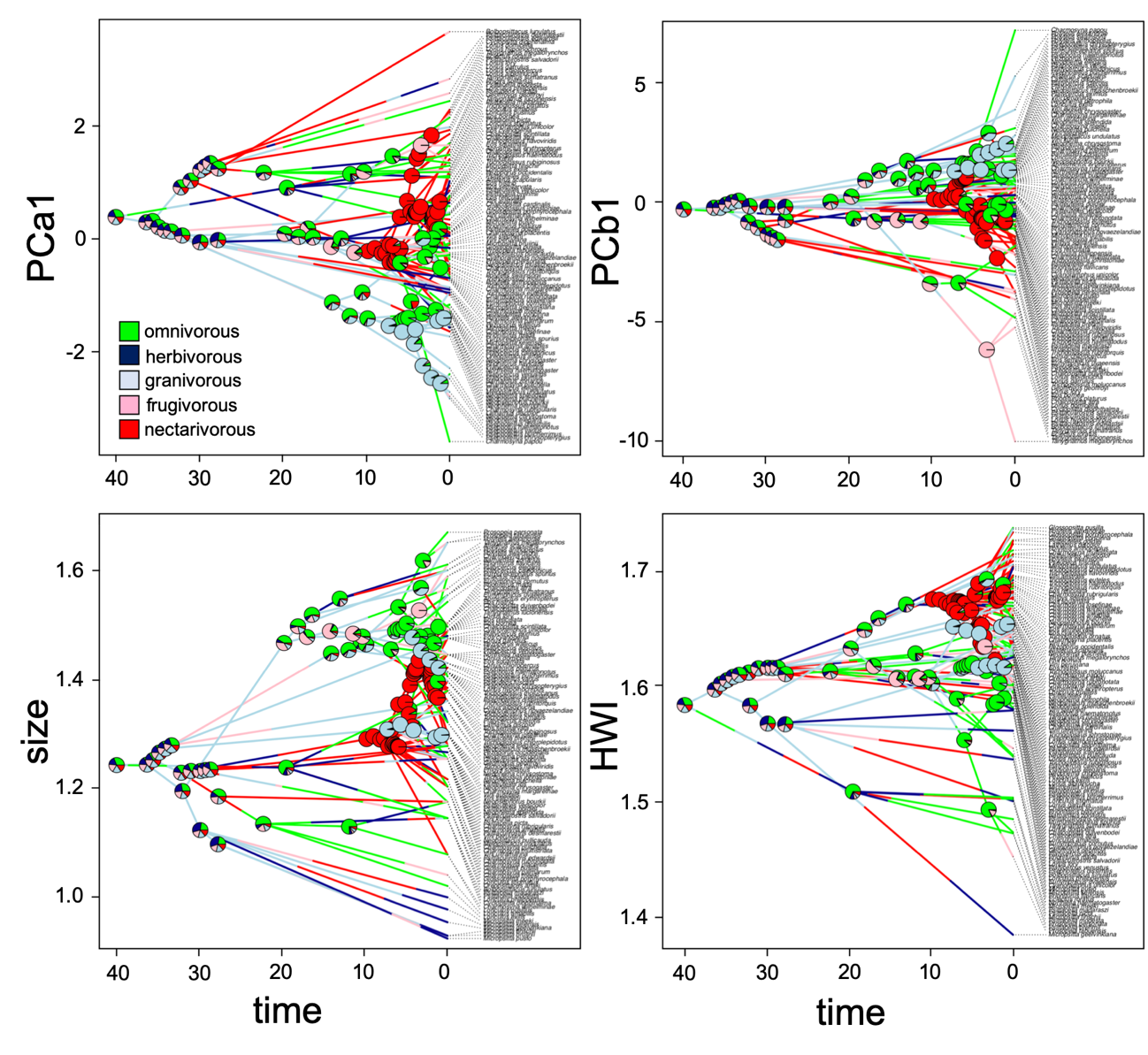


**Figure S2.** Stochastically mapped discrete character history of nectarivory in psittaculid parrots overlaid on a traitgram plot of PCa1, PCb1, body size, and HWI. The posterior probabilities from stochastic mapping are represented at each node.


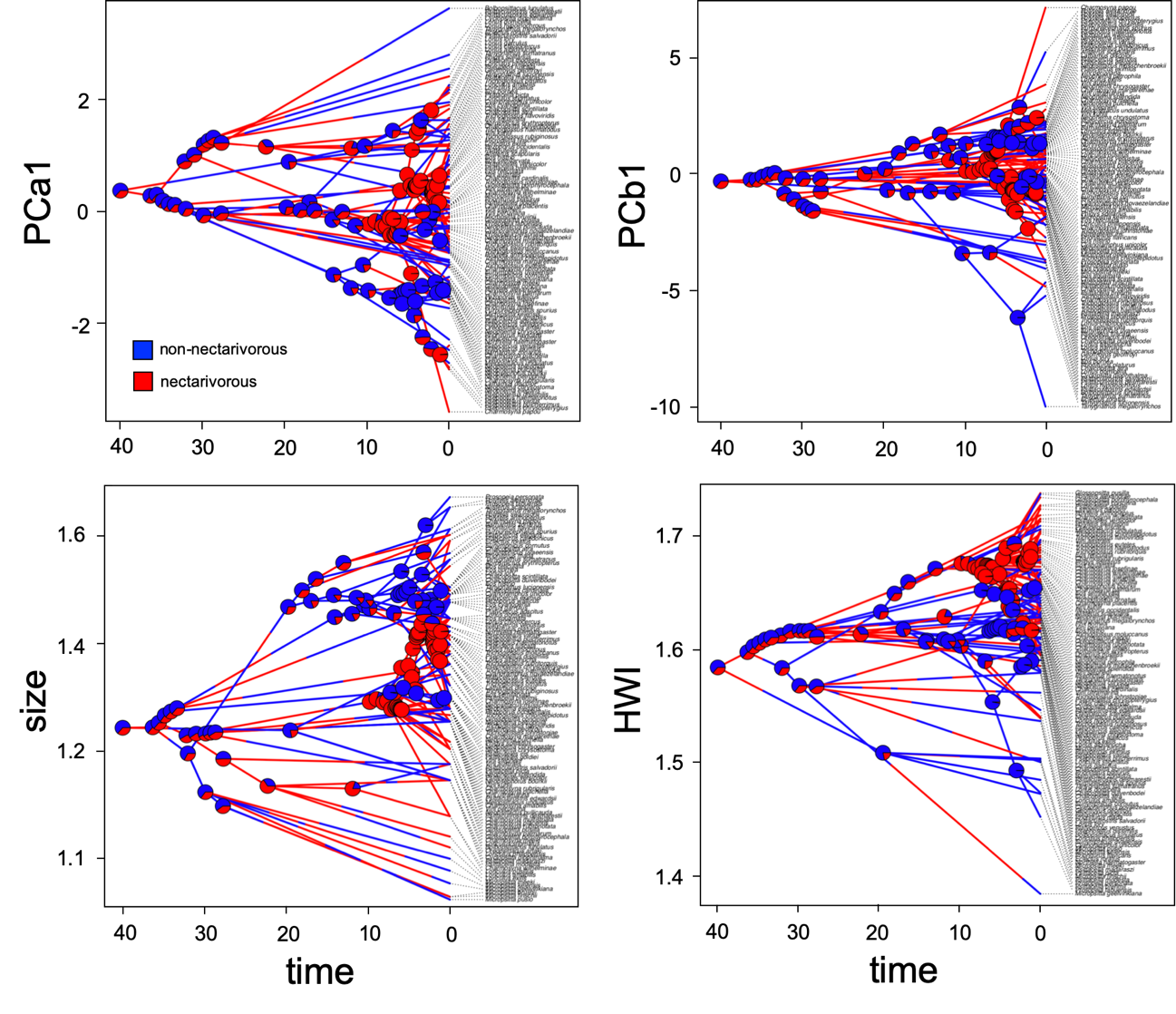


**Figure S3.** Posterior probability that a branch has a jump (left) and the posterior mean number of jumps per branch (right) on a color scale from black (no jump) to red (jumps) for each trait (*a*: body size, *b*: PCa1; *c*: PCb1; and *d*: HWI) following the approach devised by [32].

**(a)**

**
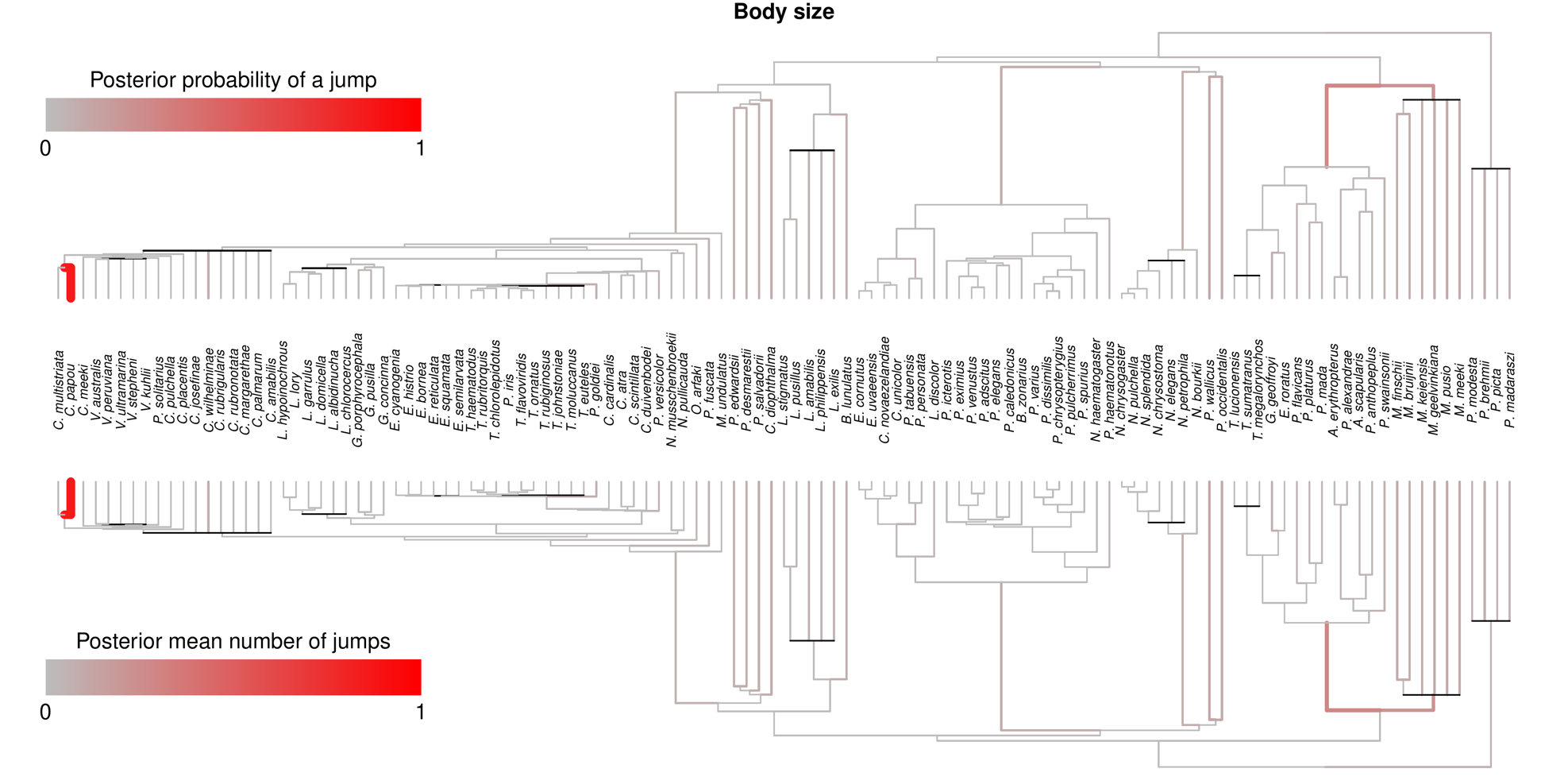
(b)**

**
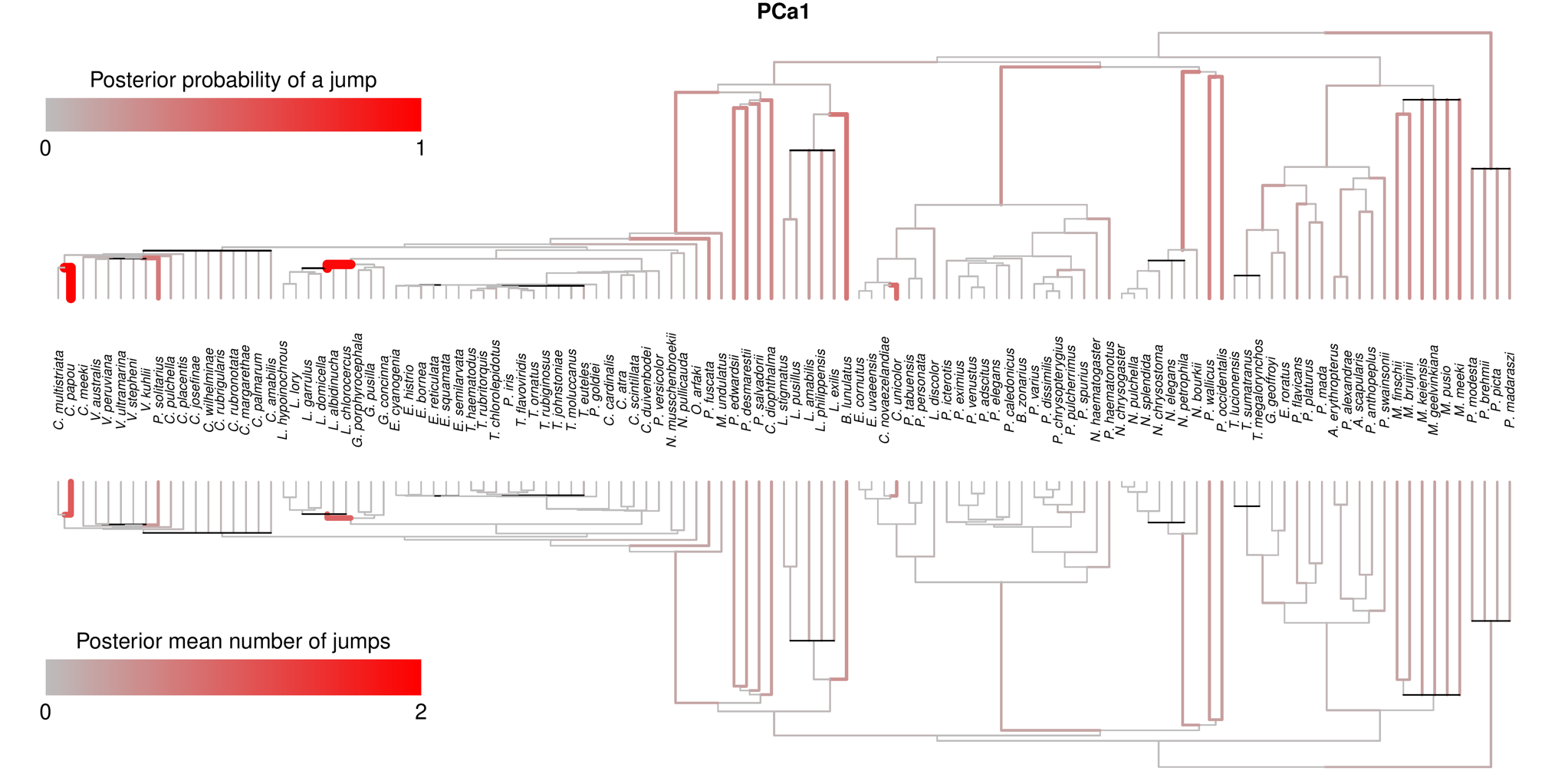
**

**(c)**

**
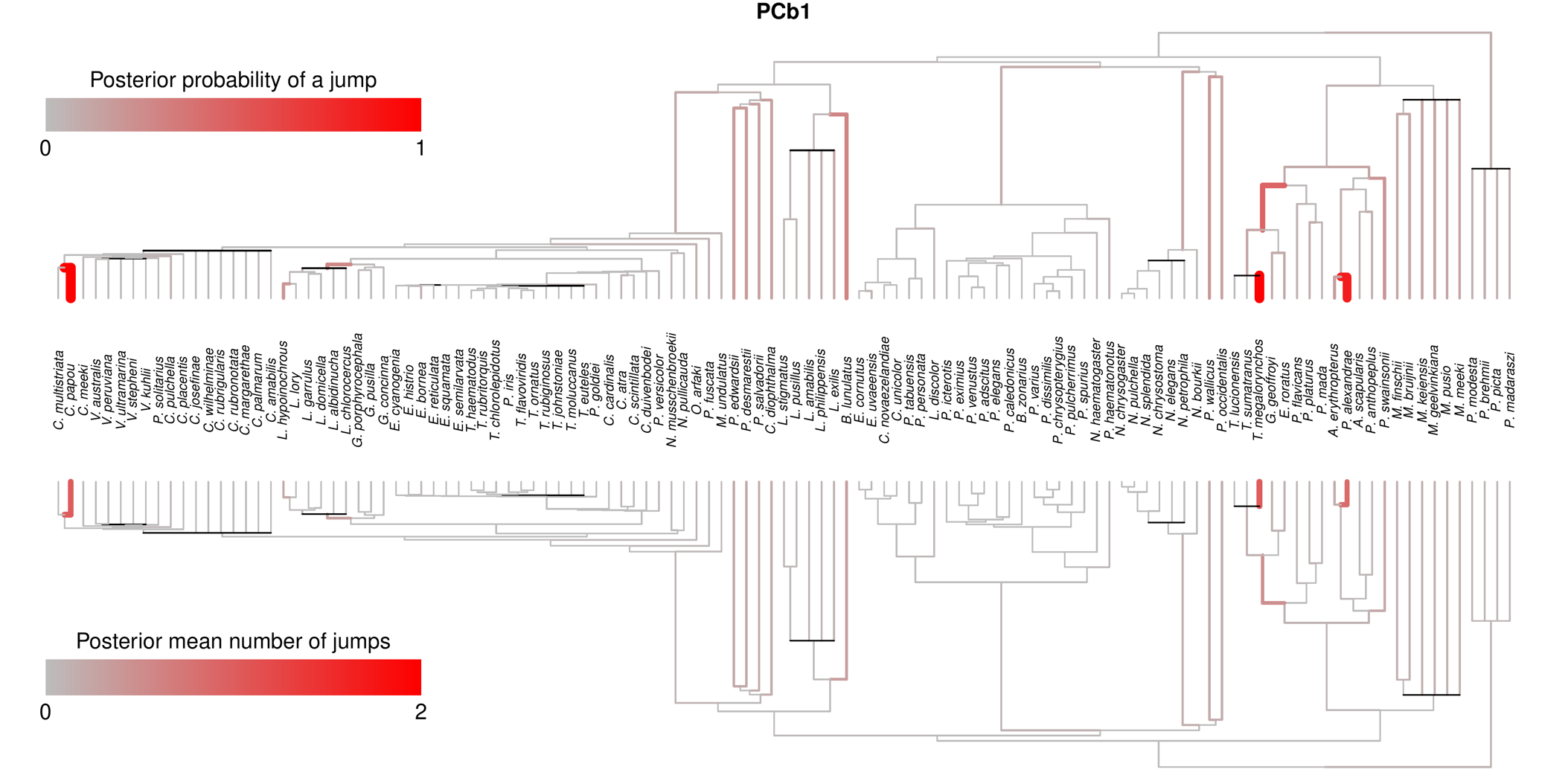
**

**(d)**

**
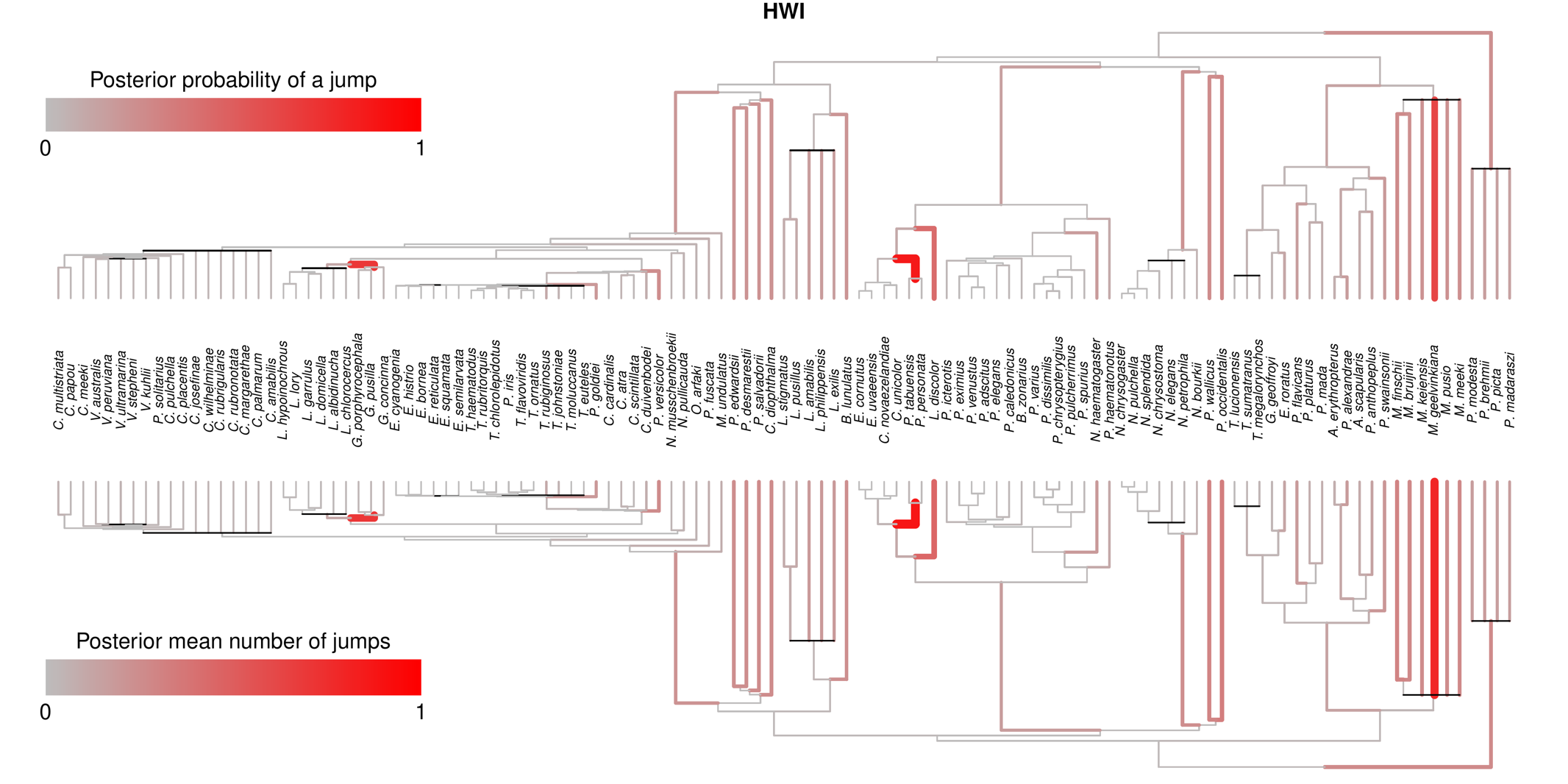
**

**Figure S4.** Rate-through-time plots for phenotypic evolution rate (with 95% confidence intervals indicated by shaded areas) in lories (Loriinae) obtained using Bayesian analysis of macroevolutionary mixtures.


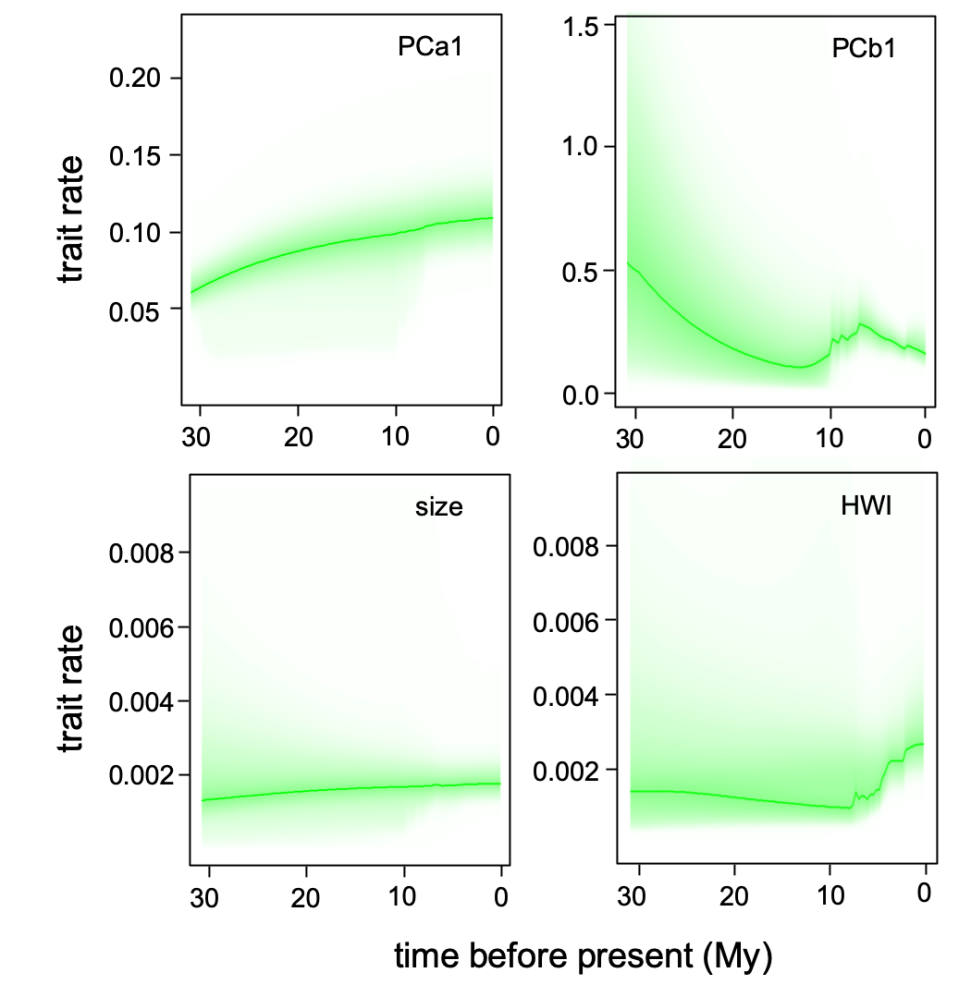


**Figure S5.** Rates of phenotypic evolution, estimated using BAMM, on different traits (a: PCa1, b: PCb1, c: body size, and d: HWI) during the radiation of Australasian psittaculid parrots. Smaller multipanel phylogenies (a-d) show the distinct rate-shift configurations with the highest posterior probability. For each distinct shift configuration, the locations of rate shifts are shown as circles, with circle size proportional to the marginal probability of the shift. Text labels (e.g., *f* = 0.25) indicate the posterior probability of each shift configuration.


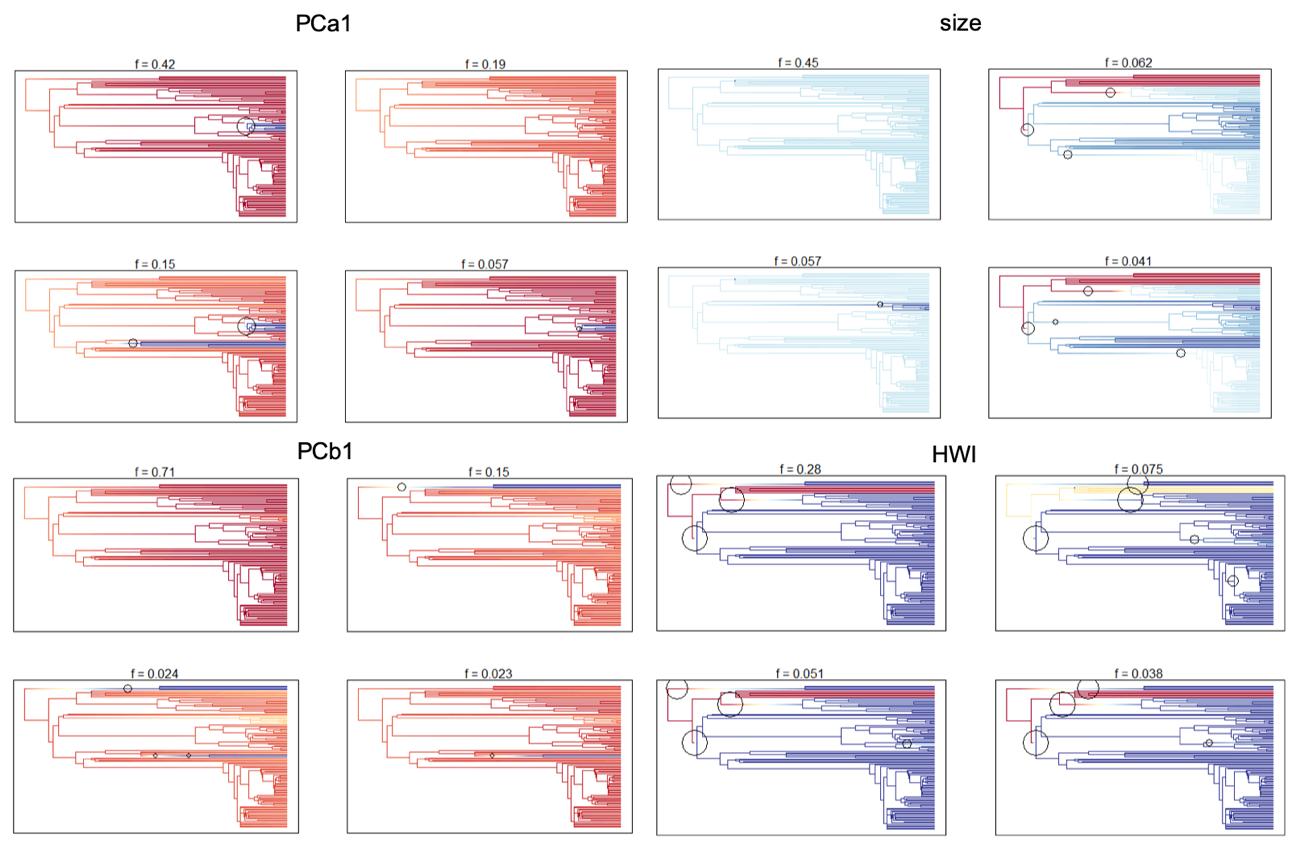

Supplement: Supplementary file 1 — Additional file 1: Table S1. Trait optima (θ) and rate of evolution or ‘drift variance’ (σ2) estimated for both categorizations (nectarivory, 2-regimes: N = nectarivorous, NN = non-nectarivorous; diet, 5-regimes: O = omnivory; F = fruits; P = plants; S = seeds; N = nectar) using the full model (OUMVA) in ‘OUwie’ (Beaulieu & O’Meara, 2015). This model all allows all parameters (θ, σ2 and α) to vary by regime. Due to its complexity the likelihood of the OUMVA model for both PCa1 and size did not converge when discerning among the five dietary regimes. It resulted in biologically unfeasible θ values and negative eigenvalues of the Hessian matrix so instead, we reported values obtained using a simpler model (OUMA), which assumes distinct θ and α for each regime while keeping constant σ2. Fig. S1. Stochastically mapped discrete character history of diet in psittaculid parrots overlaid on a traitgram plot of PCa1, PCb1, body size, and HWI. The posterior probabilities from stochastic mapping are represented at each node. Fig. S2. Stochastically mapped discrete character history of nectarivory in psittaculid parrots overlaid on a traitgram plot of PCa1, PCb1, body size, and HWI. The posterior probabilities from stochastic mapping are represented at each node. Fig. S3. Posterior probability that a branch has a jump (left) and the posterior mean number of jumps per branch (right) on a color scale from black (no jump) to red (jumps) for each trait (a: body size, b: PCa1; c: PCb1; and d: HWI) following the approach devised by [32]. Fig. S4. Rate-through-time plots for phenotypic evolution rate (with 95% confidence intervals indicated by shaded areas) in lories (Loriinae) obtained using Bayesian analysis of macroevolutionary mixtures. Fig. S5. Rates of phenotypic evolution, estimated using BAMM, on different traits (a: PCa1, b: PCb1, c: body size, and d: HWI) during the radiation of Australasian psittaculid parrots. Smaller multipanel phylogenies (a-d) show the distinct rate-shi [file 12862_2021_1940_MOESM1_ESM.docx]
